# Supplementary material for: Understanding soil selenium accumulation and bioavailability through size resolved and elemental characterization of soil extracts
Source: Nat Commun. 2022 Nov 15;13:6974. doi: 10.1038/s41467-022-34731-6 (PMC9666626; doi:10.1038/s41467-022-34731-6)
Supplement: Supplementary file 1 — Supplementary Information [file 41467_2022_34731_MOESM1_ESM.pdf]

Supplementary information

Understanding soil selenium accumulation and bioavailability through size resolved and elemental characterization of soil extracts

Julie Tolu[1, 2, *], Sylvain Bouchet[1, 2] Julian Helfenstein[3, 4], Olivia Hausheer[1, 2], Sarah Chékifi[1, 2], Emmanuel Frossard[3], Federica Tamburini[3], Oliver A. Chadwick[5], and Lenny H.E. Winkel[1, 2, *]

[1] Eawag, Swiss Federal Institute of Aquatic Science and Technology, Department of Water Resources and Drinking Water (W+T), Überlandstrasse 133, 8600 Dübendorf, Switzerland
[2] ETH Zürich, Swiss Federal Institute of Technology, Department of Environment Systems Sciences (D-USYS), Institute of Biogeochemistry and Pollutant Dynamics (IBP), Group of Inorganic Environmental Geochemistry, Universitätstrasse 16, 8092 Zürich, Switzerland
[3] ETH Zürich, Swiss Federal Institute of Technology, Department of Environment Systems Sciences (D-USYS), Institute of Agricultural Sciences (IAS), Group of Plant Nutrition, Eschikon 33, 8315 Lindau, Switzerland
[4] Present address: Wageningen University, Soil Geography and Landscape Group, 6700 AA Wageningen, The Netherlands
[5] University of California, Department of Geography, CA 93106 Santa Barbara, United States

***corresponding author:** julie.tolu@eawag.ch; lenny.winkel@eawag.ch

Contents

Supplementary Note 1. Commonly used selective extraction 3
Supplementary Note 2. Selectivity of "selective" extractions 3
Supplementary Note 3. Comparison between commonly used "selective" extractions of water-soluble and organic Se 4
Supplementary Discussion 1. Operating conditions used for the optimization of the SEC separation and of the optimal SEC separation 7
Supplementary Discussion 2. Details on the optimization of the SEC separation 10
- **a) SEC separation of (trace) elements with the 3 tested columns and optimal column selection** 10
- **b) SEC separation of (trace) elements with different NH_4NO_3 concentrations in the mobile phase and selection of the optimal NH_4NO_3 concentration** 12
- **c) SEC separation of (trace) elements with different MeOH concentrations in the mobile phase and selection of the optimal MeOH concentration** 13

Supplementary Discussion 3. Classification of elements into fractions of different size and chemical properties 14
- **a) Targeted size and size determination with the optimized SEC-UV-ICP-MS/MS method** 14
- **b) Fraction F1: (Organo)mineral nanoparticles** 15
- **c) Fractions F2 and F3: larger, more hydrophobic and/or negatively charged, OM fractions** 17
- **d) Fractions F4 and F5: Small hydrophilic compounds and free oxyanions** 18

Supplementary Discussion 4. Comparison between Se oxyanions quantified by AEC-ICP-MS/MS and SEC-UV-ICP-MS/MS 19
Supplementary Discussion 5. Stability of the SEC-ICP-MS/MS analysis during long runs 20
Supplementary Discussion 6. Reproducibility of the SEC-UV-ICP-MS/MS method for Se speciation 22
Supplementary Method 1. Obtained data for certified reference materials 28
Supplementary Method 2. Comparison of water extraction efficiencies obtained with different solid/liquid ratios 31
Supplementary Method 3. On-line isotope dilution calculation including interferences and mass bias corrections 31
Supplementary References 34

Supplementary Note 1. Commonly used selective extraction

Sequential or parallel single extraction protocols generally target an estimation of:
1) Water-soluble and plant-available Se oxyanions and amino-acids;
2) Exchangeable, mineral-adsorbed Se oxyanions; and
3) Organic Se (i.e., Se complexed or covalently bound to organic matter)

To quantify water-soluble Se, the most widely used extraction solution is ultrapure water[1–19], followed by calcium chloride ($CaCl_2$; concentration between 0.005-0.01 M)[6,20–23], 0.25 M potassium chloride (KCl)[18,24–29], 0.01 M calcium nitrate ($Ca(NO_3)_2$)[30], or 0.01 M potassium nitrate (KNO_3)[31]. Ultrapure water has also been widely used to determine solid/liquid distribution coefficients of Se, which is a parameter used to model the mobility of Se, and trace elements in general, in soils[(e.g., 32,33)].

To quantify exchangeable, mineral-adsorbed Se oxyanions, the most widely used extraction solution is potassium phosphate (concentration between 0.016 and 0.1 M; pH 7 or 8)[3,5–12,15–20,24–31,34–38], followed by ammonium oxalate (pH 3)[21,22], or ammonium chloride[14].

To quantify organic Se, the most widely used extraction solution is 0.1 M NaOH[1–4,6,9,10,14,15,17,20,21,39,34,40], probably because this extraction solution has been widely used to extract and characterize soil organic matter and associations of trace elements with organic matter (see review of Olk et al.[41]). Other studies replaced 0.01 M NaOH by NaOCl[12,21,26,35], $K_2S_2O_8$[3,11,19,25,37,38], a mixture of pyrophosphate/NaOH[42,43], NH_4OH[18], or TMAH[31].

Supplementary Note 2. Selectivity of "selective" extractions

The lack in selectivity of "selective" extraction procedures mainly come from the used extraction solutions, although the extraction conditions can lead to incomplete extractions of targeted Se species (e.g., too short extraction time or too small solid:liquid ratios) or can lead to contamination or Se species transformation (e.g., with sequential versus parallel single extractions)[4,12,19,44].

While Se extracted by ultrapure water, $CaCl_2$, KCl, $Ca(NO_3)_2$ and KNO_3 is assumed to include Se(IV), Se(VI), and seleno-amino acids, few studies have shown that seleno-amino acids are absent or present in very low amount in ultrapure water or $CaCl_2$ extracts and that large proportions of Se in these extracts do not eluted from anion exchange chromatography (AEC)[6,9,10,36]. These results imply that large proportions of Se in these extracts is present in other forms than Se(VI), Se(IV), and seleno-amino acids and so water-soluble Se cannot be considered as representative of bioavailable free Se oxyanions and seleno-amino acids. In addition, correlations were found between the concentrations of total Se or AEC-unidentified Se in water or $CaCl_2$ extracts and the concentrations of organic carbon in these extracts[10,21]. Weng et al.[23] also showed by applying donnan-membrane technology to $CaCl_2$ soil extracts that large proportion of Se in $CaCl_2$ extracts was of colloidal forms. Overall, these previous studies suggest that water, $CaCl_2$ and KCl extracts contain large amount of organic Se (other than seleno-amino acids), Se associated to (organo)mineral nanoparticles and/or Se(0) nanoparticles.
Concerning step 2 and 3, phosphate buffer extractions were shown to incompletely extract mineral-adsorbed Se oxyanions[12,44], while NaOH was demonstrated to extract mineral-adsorbed Se(IV) that was not extracted by phosphate buffers[44]. The higher efficiency of NaOH to extract mineral-adsorbed Se(IV) with respect to phosphate buffers is due to both the competition of OH- with Se(IV) for mineral surfaces

and the higher pH of 0.1 M NaOH solutions (pH 14 versus 7-8 for phosphate buffers) leading to negative surface charges of the (oxy)hydroxides that impede re-adsorption of desorbed Se(IV)[44]. On another hand, phosphate buffers were suggested to extract some organic Se forms because large proportions of Se species (up to 43%) remain unidentified in phosphate extracts using AEC-ICP-MS/MS (i.e., are not found as Se oxyanions)[6,9,10,36] and large proportions of soil organic carbon is solubilized in phosphate extracts (7-38%, n=26 soils)[9]. Using ammonium oxalate instead of phosphate buffers to extract exchangeable, mineral-adsorbed Se oxyanions seems to be associated with the same selectivity issues; for examples, as in phosphate extracts, large proportions of Se exist in other forms than Se oxyanions in ammonium oxalate extracts, which solubilize 4-12% of soil organic carbon in agricultural soils[21]. Concerning step 3, as with NaOH, NaOCl and $K_2S_2O_8$ extract mineral adsorbed Se(IV) that is not extracted by phosphate buffer, but also oxidize part of Se(0) (nano)particles and metal selenides[12,19]. In line with this, a previous studies reported higher amount of soil Se extracted with NaOCl than with NaOH[21]. On another hand, the selectivity of NH_4OH[(e.g., 18)] and TMAH[(e.g., 31)] solutions for targeted organic Se has never been tested. However, given the high concentrations of OH^- groups and the alkaline pH of these extraction solutions, it can be assumed that NH_4OH[(e.g., 18)] and TMAH[(e.g., 31)] extract, in addition to organic Se, mineral adsorbed Se(IV) that is not extracted by the phosphate buffer step as when using NaOH.
Overall, all these different examples clearly show that quantifying total Se in soil extracts and assuming that it represents one or certain Se species is inaccurate and so having a comprehensive determination of Se speciation in soil extracts is necessary to understand soil Se accumulation in soils and plant-availability**.**

Supplementary Note 3. Comparison between commonly used "selective" extractions of water-soluble and organic Se

Although different extraction solutions (cf. Supplementary Note 1 above) and different parameters (solid/liquid ratios, extraction times, and investigated soils) were used, similar ranges in extraction efficiencies were overall reported. Water-soluble Se was estimated, based on extractions with ultrapure water[1–18], $CaCl_2$[6,20–23], KCl[18,24–29], $Ca(NO_3)_2$[30], and KNO_3[31] to account for ~0.1 up to ~20% of total soil Se. Organic Se was estimated based on extractions with 0.1 M NaOH[1–4,6,9,10,14,15,17,20,21,39,34,40], NaOCl[12,21,26,35], $K_2S_2O_8$[3,11,25,37,38], pyrophosphate/NaOH mixture[42,43], NH_4OH[18], and TMAH[31], to account for ~20 up to ~80% of total soil Se.

Additionally, in a previous study, Se extraction efficiencies obtained with ultrapure water and 0.005 M $CaCl_2$ were compared for one soil and were shown to be very similar, i.e., 1.4±0.2 and 0.9±0.1 % of total soil Se for ultrapure water and $CaCl_2$, respectively[6]. Moreover, the proportions of Se species that are not present as Se(IV) and Se(VI) (which were determined by AEC-ICP-MS/MS) in the ultrapure water extracts of Kohala soils are highly similar to those found by Weng et al.[23], who used 0.01 M $CaCl_2$ instead of ultrapure water, i.e., respectively, 43-100 % of total Se extracted by water (n= 25 Kohala soils) and 67-86 % of total Se extracted by $CaCl_2$ (n=15 grassland soils from the Netherlands).

Supplementary Table 1. Location and additional information on the six study Kohala sites (S1-S6)

	**Elevation (m above sea level)**	**Distance from site S1 (km)**	**Precipitation (mm year^{-1})**	**Longitude (° west)**	**Latitude (° north)**	**Sampled depth**	**Soil horizon of samples depth**
**S1**	70	0.0	275	-155.8798	20.1132	Up to 20cm (every 10cm)	0-10cm: horizon A: 10-20cm: horizon B
**S2**	128	0.7	316	-155.8737	20.1155	Up to 20cm (every 10cm)	0-10cm: horizon A: 10-20cm: horizon B
**S3**	619	6.4	1340	-155.8208	20.1515	Up to 50cm (every 10cm)	0-20cm: horizon A: 20-50cm: horizon B
**S4**	735	7.5	1578	-155.8307	20.1480	Up to 70cm (every 10cm)	0-30cm: horizon A: 30-70cm: horizon B
**S5**	860	10.1	2163	-155.7485	20.1347	Up to 40cm (every 10cm)	0-20cm: horizon A: 20-40cm: horizon B
**S6**	1059	15.6	3123	-155.7972	20.1555	Up to 50cm (every 10cm)	0-10cm: horizon A: 10-50cm: horizon B

Supplementary Table 2. Soil properties along the Kohala climate gradient

Site		rainfall (mm yr^{-1})	pH	SOC[a] (%)	TON_{soil}[b] (%)	C/N_{soil}[c]	Al_{soil}[d] (%)	Fe_{soil}[e] (%)	Mn_{soil}[f] (g kg^{-1})	$Fe_{amorphous}$[g] (g kg^{-1})	$Fe_{crystalline}$[h] (g kg^{-1})	Se_{soil}[i] (mg kg^{-1})	As_{soil}[j] (mg kg^{-1})	S_{soil}[k] (g kg^{-1})
**S1**	**av**[l] ± sd[m]	275	**7.1** ± 0.5	**2** ± 2	**0.2** ± 0.1	**10** ± 2	**3.9** ± 0.2	**13.8** ± 0.1	**2.9** ± 0.2	**10.0** ± 0.3	**38.2** ± 0.2	**1.2** ± 1.1	**3.7** ± 0.9	**1.0** ± 1.1
	min[n] *– max*[o]		*6.7 – 7.4*	*1 – 4*	*0.1 – 0.3*	*9 – 12*	*3.8 – 4.1*	*13.7 – 13.8*	*2.7 – 3.0*	*9.8 – 10.2*	*38.1 – 38.3*	*0.4 – 1.9*	*3.0 - 3.2*	*0.2 – 1.7*
**S2**	**av**[l] ± sd[m]	316	**6.5** ± 0.1	**3** ± 1	**0.3** ± 0.1	**12** ± 1	**4.3** ± 0.2	**13.0** ± 0.2	**2.9** ± 0.1	**9** ± 1	**34.9** ± 0.9	**0.8** ± 0.4	**3.4** ± 0.2	**0.9** ± 0.6
	min[n] *– max*[o]		*6.4 – 6.5*	*2 – 4*	*0.2 – 0.3*	*11 – 12*	*4.1 – 4.4*	*12.8 – 13.1*	*2.8 – 2.9*	*9 – 10*	*34.2 – 35.5*	*0.6 – 1.1*	*3.5– 4.3*	*0.5 – 1.3*
**S3**	**av**[l] ± sd[m]	1340	**6.5** ± 0.2	**4** ± 3	**0.4** ± 0.3	**9.2** ± 0.4	**2.1** ± 0.6	**8.6** ± 0.5	**7** ± 1	**34** ± 4	**19** ± 4	**0.6** ± 0.4	**5.0** ± 0.9	**1.4** ± 0.3
	min[n] *– max*[o]		*6.3 – 6.6*	*1 – 9*	*0.1 – 1.0*	*8.8 – 9.6*	*1.6 – 3.1*	*7.8 – 9.0*	*6 – 8*	*31 – 37*	*16 – 22*	*0.2 – 1.1*	*3.6 – 5.7*	*1.2 – 1.6*
**S4**	**av**[l] ± sd[m]	1577	**6.2** ± 0.4	**6** ± 1	**0.6** ± 0.1	**9.9** ± 0.5	**1.4** ± 0.2	**9** ± 1	**4.1** ± 0.6	**28** ± 5	**24** ± 8	**1.1** ± 0.3	**7.6** ± 0.1	**0.7** ± 0.3
	min[n] *– max*[o]		*6.9 – 6.5*	*4 – 7*	*0.4 -. 0.7*	*9.4 – 10.8*	*1.1 – 1.7*	*8 – 11*	*3.3 – 4.9*	*24 – 31*	*19 – 30*	*0.7 – 1.5*	*7.4 – 7.8*	*0.5 – 1.0*
**S5**	**av**[l] ± sd[m]	216	**5.2** ± 0.3	**13** ± 4	**1.0** ± 0.5	**14** ± 2	**0.9** ± 0.1	**14** ± 2	**0.5** ± 0.1	**72** ± 11	**27** ± 4	**2.6** ± 0.4	**16** ± 1	**0.6** ± 0.3
	min[n] *– max*[o]		*5.0 – 5.4*	*10 – 19*	*0.6 – 1.6*	*12 – 16*	*0.9 - 1.0*	*12 – 15*	*0.4 – 0.6*	*64 – 79*	*25 – 30*	*2.3 – 3.1*	*14 – 17*	*0.4 – 0.8*
**S6**	**av**[l] ± sd[m]	3123	**4.4** ± 0.3	**16** ± 6	**1.1** ± 0.6	**16** ± 2	**0.7** ± 0.1	**4.5** ± 0.9	**0.5** ± 0.2	**12** ± 2	**5** ± 3	**2.6** ± 0.7	**6** ± 1	**0.89** ± 0.04
	min[n] *– max*[o]		*4.2 – 4.6*	*12 – 26*	*0.7 – 2.2*	*12 - 18*	*0.5 – 0.9*	*3.6 – 6.0*	*0.4 – 0.8*	*11 - 13*	*2 - 7*	*2.0 – 3.6*	*4 – 7*	*0.87 – 0.92*

[a]SOC: soil organic carbon concentration; [b]TON_{soil}: total organic nitrogen concentration in soil; [c]C/N_{soil}: total carbon to total nitrogen ratio in soil; [d]Al_{soil}: total aluminum concentration in soil; [e]Fe_{soil}: total iron concentration in soils; [f]Mn_{soil}: total manganese concentration in soil; [g]$Fe_{amorphous}$: concentration of amorphous Fe (oxy)hydroxides in soil estimated from the Fe concentration in oxalate extracts; [h]$Fe_{crystalline}$: concentration of crystalline Fe (oxy)hydroxides estimated from the difference between the Fe concentration in dithionite-citrate bicarbonate extracts and the Fe concentrations in the oxalate extracts; [i]Se_{soil}: total selenium concentration in soil; [j]As_{soil}: total arsenic concentration in soil; [k]S_{soil}: total sulfur concentration in soil. [l]av: average of all analyzed soil horizon samples (for pH, $Fe_{amorphous}$ and $Fe_{crystalline}$) or soil depth samples (for all other parameters) considering the average value obtained for each sample (cf. note below) ; [m]sd: standard deviation for all analyzed soil horizon samples (for pH, $Fe_{amorphous}$ and $Fe_{crystalline}$) or soil depth samples (for all other parameters) considering the average value obtained for each sample; [n]min: minimal value among all analyzed soil horizon samples (for pH, $Fe_{amorphous}$ and $Fe_{crystalline}$) or soil depth samples (for all other parameters) considering the average value obtained for each sample; [o]max: maximal value among all analyzed soil horizon samples (for pH, $Fe_{amorphous}$ and $Fe_{crystalline}$) or soil depth samples (for all other parameters) considering the average value obtained for each sample.

Note: the concentrations of pH, $Fe_{amorphous}$ and $Fe_{crystalline}$ were measured by Helfenstein et al.[45] on sampled soil horizon A and B without triplicate. The concentrations of SOC, TON_{soil}, Al_{soil}, Fe_{soil}, Mn_{soil}, Se_{soil}, As_{soil}, and S_{soil} were determined in triplicate for each analyzed soil depth samples.

Supplementary Discussion 1. Operating conditions used for the optimization of the SEC separation and of the optimal SEC separation

A complete description of the operating conditions used for the optimization of the SEC separation and for the analysis of all Kohala soil extracts is given in Supplementary Table 3 and associated footnotes. In brief, the optimization of the SEC separation was achieved using extracts from the contrasting topsoils (0-10 cm) of Kohala sites S1, S4, and S6 as follows:

1) Optimization phase 1: We tested three SEC columns containing different stationary phases (in terms of size range and materials), which were previously used to investigate trace element speciation or OM size and composition in natural waters or reference humic substances materials[46–53]. We, however, only selected columns that tolerate alkaline mobile phases, necessary to analyze NaOH extracts (i.e., silica based columns were not tested).

2) Optimization phase 2: We tested different ammonium nitrate (NH_4NO_3; used as salt) concentrations in the mobile phase using the column selected in optimization phase 1.

3) Optimization phase 3: We tested different methanol (MeOH) concentrations in the mobile phase using the column selected in optimization phase 1 and the mobile phase NH_4NO_3 concentration selected in optimization phase 2.

4) Selection of the optimal SEC separation: the selection of the optimal SEC column and mobile phase composition to determine Se speciation in soil extracts was based on the resolution of the SEC separation of Se and other trace elements, on the SEC recovery of Se species, and on the capability to detect total C with ICP-MS/MS. The selection of the optimal SEC conditions is described in detailed in Supplementary Discussion 2 thereafter.

5) Final analysis of all Kohala soil extracts: the analysis of all soil extracts was carried out using the optimal SEC method, which involves a series of Shodex OH-pak SB-803 and -802.5 HQ columns and a mobile phase containing 5 mmol L^{-1} NH_4NO_3 at pH 7.5 and pH 9 for water and NaOH extracts, respectively.

Supplementary Table 3. Operating conditions for the optimization of the SEC separation and the analysis of all Kohala soil extracts with the optimized SEC

	Optimization phase 1	Optimization phase 2	Optimization phase 3	Optimal method
**Analyzed soil extracts**	Water and NaOH extracts from topsoils (0-10 cm) of Kohala sites S1, S4, and S6[(a)]	Water and NaOH extracts from topsoils (0-10 cm) of Kohala sites S1, S4, and S6[(a)]	Water and NaOH extracts from topsoils (0-10 cm) of Kohala sites S1, S4, and S6[(a)]	Water and NaOH extracts from all Kohala soils (n=25)
**SEC column(s)**	- Superdex peptide 10/300 GL[(b)]<br>- PL-aquagel-OH 30[(b)]<br>- OH-pak SB-803-802.5HQ series[(b)]	OH-pak SB-803-802.5HQ series[(b)]	OH-pak SB-803-802.5HQ in series[(b)]	OH-pak SB-803-802.5HQ series[(b)]
**Mobile phase composition**				
Salt concentrations	5 mmol L^{-1} NH_4NO_3[(c)]	5, 20 and 50 mmol L^{-1} NH_4NO_3[(c, d)]	5 mmol L^{-1} NH_4NO_3[(c)]	5 mmol L^{-1} NH_4NO_3[(c)]
MeOH concentrations	0% MeOH	0% MeOH	0, 10 and 20% MeOH[(d)]	0% MeOH
pH (for each extract type)	7 (water); 9.5 (NaOH)[(e)]	7 (water); 9.5 (NaOH)[(e)]	7 (water); 9.5 (NaOH)[(e)]	7 (water); 9.5 (NaOH)[(e)]
**Mobile phase flow rate**	1 mL min^{-1}	1 mL min^{-1}	1 mL min^{-1}	1 mL min^{-1}
**Sample injection volume**	100 µL	100 µL	100 µL	100 µL
**On-line internal standard**	^{77}Se(IV) (5 µg L^{-1}), ^{45}Sc (70 µg L^{-1}), ^{89}Y (70 µg L^{-1})[(f)]	^{77}Se(IV) (5 µg L^{-1}), ^{45}Sc (70 µg L^{-1}), ^{89}Y (70 µg L^{-1})[(f)]	^{77}Se(IV) (5 µg L^{-1}), ^{45}Sc (70 µg L^{-1}), ^{89}Y (70 µg L^{-1})[(f)]	^{45}Sc (70 µg L^{-1}), ^{89}Y (70 µg L^{-1})[(f)]
**On-line isotope dilution**	No	No	No	Yes, with ^{78}Se(IV)[(g)]
**UV detection**	No	No	No	Wavelength of 254 nm
**ICP-MS/MS detection 1**				
Gas in collision/reaction cell	5 mL min^{-1} H_2	5 mL min^{-1} H_2	5 mL min^{-1} H_2	5 mL min^{-1} H_2
Acquired isotopes and acquisition time	Selenium (Se): *m/z* 77-77, 78-78, 80-80; 0.3 ms<br>Arsenic (As): *m/z* 75-75; 0.3 ms<br>Iron (Fe): *m/z* 56-56; 0.1 ms<br>Copper (Cu): *m/z* 65-65; 0.1 ms<br>Zinc (Zn): *m/z* 66-66; 0.1 ms<br>Lead (Pb): *m/z* 208-208; 0.1 ms<br>Scandium (Sc): *m/z* 45-45; 0.1 ms<br>Yttrium (Y): *m/z* 89-89; 0.1 ms	Selenium (Se): *m/z* 77-77, 78-78, 80-80; 0.3 ms<br>Arsenic (As): *m/z* 75-75; 0.3 ms<br>Iron (Fe): *m/z* 56-56; 0.1 ms<br>Copper (Cu): *m/z* 65-65; 0.1 ms<br>Zinc (Zn): *m/z* 66-66; 0.1 ms<br>Lead (Pb): *m/z* 208-208; 0.1 ms<br>Scandium (Sc): *m/z* 45-45; 0.1 ms<br>Yttrium (Y): *m/z* 89-89; 0.1 ms	Selenium (Se): *m/z* 77-77, 78-78, 80-80; 0.3 ms<br>Arsenic (As): *m/z* 75-75; 0.3 ms<br>Iron (Fe): *m/z* 56-56; 0.1 ms<br>Copper (Cu): *m/z* 65-65; 0.1 ms<br>Zinc (Zn): *m/z* 66-66; 0.1 ms<br>Lead (Pb): *m/z* 208-208; 0.1 ms<br>Scandium (Sc): *m/z* 45-45; 0.1 ms<br>Yttrium (Y): *m/z* 89-89; 0.1 ms	Selenium (Se): *m/z* 77-77, 78-78, 80-80; 0.3 ms<br>Arsenic (As): *m/z* 75-75; 0.3 ms<br>Iron (Fe): *m/z* 56-56; 0.1 ms<br>Copper (Cu): *m/z* 65-65; 0.1 ms<br>Zinc (Zn): *m/z* 66-66; 0.1 ms<br>Lead (Pb): *m/z* 208-208; 0.1 ms<br>Scandium (Sc): *m/z* 45-45; 0.1 ms<br>Yttrium (Y): *m/z* 89-89; 0.1 ms
**ICP-MS/MS detection 2**				
Gas in collision/reaction cell	25% O_2 and 1 mL min^{-1} H_2	25% O_2 and 1 mL min^{-1} H_2	25% O_2 and 1 mL min^{-1} H_2	25% O_2 and 1 mL min^{-1} H_2
Acquired isotopes and acquisition time	Se: *m/z* 77-93, 78-94, 80->96; 0.3 ms<br>Sulfur (S): *m/z* 32->48; 0.1 ms<br>Carbon (C): *m/z* 12->28; 0.3 ms<br>Titanium (Ti): *m/z* 47->63; 0.1 ms<br>Scandium (Sc) *m/z* 45-61; 0.1 ms<br>Yttrium (Y): *m/z* 89-105; 0.1 ms	Se: *m/z* 77-93, 78-94, 80->96; 0.3 ms<br>Sulfur (S): *m/z* 32->48; 0.1 ms<br>Carbon (C): *m/z* 12->28; 0.3 ms<br>Titanium (Ti): *m/z* 47->63; 0.1 ms<br>Scandium (Sc) *m/z* 45-61; 0.1 ms<br>Yttrium (Y): *m/z* 89-105; 0.1 ms	Se: *m/z* 77-93, 78-94, 80->96; 0.3 ms<br>Sulfur (S): *m/z* 32->48; 0.1 ms<br>Carbon (C): *m/z* 12->28; 0.3 ms<br>Titanium (Ti): *m/z* 47->63; 0.1 ms<br>Scandium (Sc) *m/z* 45-61; 0.1 ms<br>Yttrium (Y): *m/z* 89-105; 0.1 ms	Se: *m/z* 77-93, 78-94, 80->96; 0.3 ms<br>Sulfur (S): *m/z* 32->48; 0.1 ms<br>Carbon (C): *m/z* 12->28; 0.3 ms<br>Titanium (Ti): *m/z* 47->63; 0.1 ms<br>Scandium (Sc) *m/z* 45-61; 0.1 ms<br>Yttrium (Y): *m/z* 89-105; 0.1 ms
**SEC-peak deconvolution**	No	No	No	for ^{78}Se, ^{80}Se, ^{75}As, and ^{32}S[(h)]

Footnotes are given on next page

Footnotes of Supplementary Table 3

(a) These 3 selected Kohala soils cover a large range in total soil Se (0.4-2.1 mg kg^{-1}), pH (4.2-7), total soil Fe (4-14%), and SOC (1-26%);

(b) The columns manufacturers and dimensions are as follows: Superdex peptide 10/300 GL (GE Healthcare; 10x300 mm; 7 µm); Aquagel-OH 30 (Agilent, 7.5x300 mm, 8 µm); and series of OH-pak SB-803HQ and SB-802.5HQ (both Shodex; 8x300 mm, 6 µm);

(c) NH_4NO_3 was selected in lieu of ammonium citrate in order to reduce carbon (C) background and thus to be able to detect C simultaneously to Se and other elements. Furthermore, NH_4NO_3 is superior to ammonium phosphate in terms of maintaining optimal sensitivity (phosphate precipitating on the ICP-MS cones);

(d) Testing the influence of different mobile phase compositions on the SEC separation of Se were done within the same day;

(e) The pH of the mobile phase was fixed to 9.5 for NaOH extracts and to 7 for the water extracts to match as best as possible the pH of the soil extracts considering the pH limitations of the selected columns;

(f) Internal standard was added post-UV detector through a T-connector and the ICP-MS/MS peristaltic pump during the optimization and the final run of all samples to account or check for changes in sensitivity. ^{77}Se(0) was purchased from Isoflex (USA), oxidized to ^{77}Se(IV) according to the procedure of Dael et al.[54], and the resulting 1000 mg L^{-1} ^{77}Se(IV) stock solution was stored at 4°C;

(g) $^{78}SeO_3^{2-}$(IV) standard was added post UV-detector to enable the quantification of Se in SEC-peaks by on-line Se isotope dilution (ID), based on conditions adapted from Sariego Muňiz et al.[55] for the modified ^{80}Se/^{78}Se isotopic ratio. The resulting Se mass flow chromatogram is then deconvoluted to obtain the Se amount in each SEC-peak, from which the Se concentrations is obtained considering the sample injection volume. The 78SeO32-(IV) standard (stock solution, 1000 mg L-1) was prepared from 78Se(0) (Isoflex, USA) according to Dael et al.[54], and was then stored at 4°C. This standard was composed of 0.0063±0.0001% of ^{74}Se, 0.003±0.001% of ^{76}Se, 0.16±0.04% of ^{77}Se, 99.50±0.02% of ^{78}Se, 0.33±0.01% of ^{80}Se and 0.008±0.004% of ^{82}Se (determined by ICP-MS/MS), and only consisted in SeO_3^{2-}(IV) (determined by AEC-ICP-MS/MS). The concentration of $^{78}SeO_3^{2-}$(IV) added post-UV detector was adjusted for all samples to reach an optimal ^{80}Se/^{78}Se ratio during the SEC-elution in order to obtain minor error propagation during ID calculation[56]. Before the ID calculation, ^{78}Se and ^{80}Se intensities were corrected for respectively, $^{1}H^{77}$Se and $^{1}H^{79}$Br interferences and the resulting ratio ^{80}Se/^{78}Se was corrected for mass bias, as described elsewhere[10];

(h) Peak deconvolution of the Se mass flow chromatograms (resulting from the isotope dilution calculation) and of the S and As intensity chromatograms was performed using the peak analyzer function (Fit peak-pro) of Origin2018 software as reported in Laborda et al.[57]. The r^2 of the peak-fit were ≥0.90 for all deconvoluted chromatograms and in most cases ≥0.96

Supplementary Discussion 2. Details on the optimization of the SEC separation

a) SEC separation of (trace) elements with the 3 tested columns and optimal column selection

Supplementary Figure 1 provides the chromatograms of Se and other elements obtained with the three tested columns for the NaOH and water extracts of Kohala S6 topsoil.

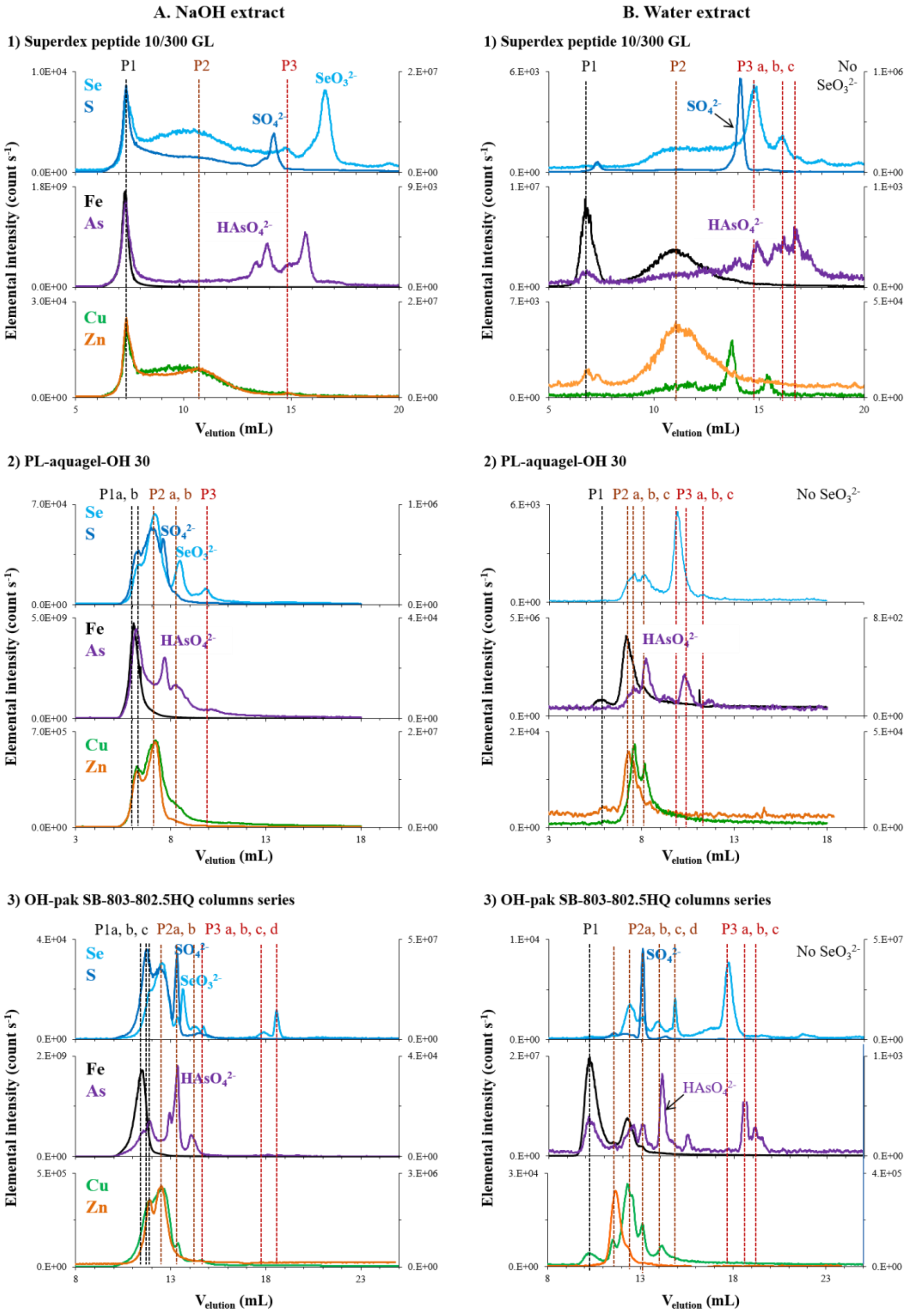

Supplementary Figure 1. Comparison of chromatograms obtained for selenium (Se), sulfur (S), iron (Fe), arsenic (As), copper (Cu), and zinc (Zn) in the NaOH (panel A) and ultrapure water (panel B) extracts of the topsoil from Kohala site S6 with the three tested SEC columns. The three tested SEC were: 1) Superdex peptide 10/300 GL; 2) PL-aquagel-OH 30; and 3) OH-pak SB-803-802.5HQ columns series. The Y-axis represents the element intensity (in count s^{-1}).

The least good resolution of the chromatographic separation was obtained with the Superdex peptide 10/300 GL column for both types of extracts. With this column, the first Se-peak in NaOH extracts co-eluted with all elements (peak noted P1 in Supplementary Figure 1; apex at ~7.5 mL). In contrast, with the PL-aquagel OH30 and the OH-pak SB-803-802.5HQ columns series, the first Se-peak is clearly separated from one or two preceding peaks (see peaks P1a, b and/or c). The size calibrations with pullulan and polystyrene sulfonate (PSS) molecular weight standards, shown in Supplementary Figure 2 (panel A), further indicate that the first elemental peak (P1 at 7.3 mL) obtained with the Superdex peptide 10/300 GL column eluted in the dead volume, and was thus larger than ~15-20 kDa. Moreover, with the Superdex peptide 10/300 GL column, most of Se and other elements (e.g., S, Cu) elute in a very broad peak (from ~8 to 15.5 mL) in both types of extracts. Such large peaks present two disadvantages: i) they lower the detection limit; and ii) their deconvolution (to obtain their peak area) is difficult or even impossible, impeding quantification of the elements in SEC-peaks (by on-line isotope dilution) or semi-quantification of the elements (using peak area normalized by an internal standard).

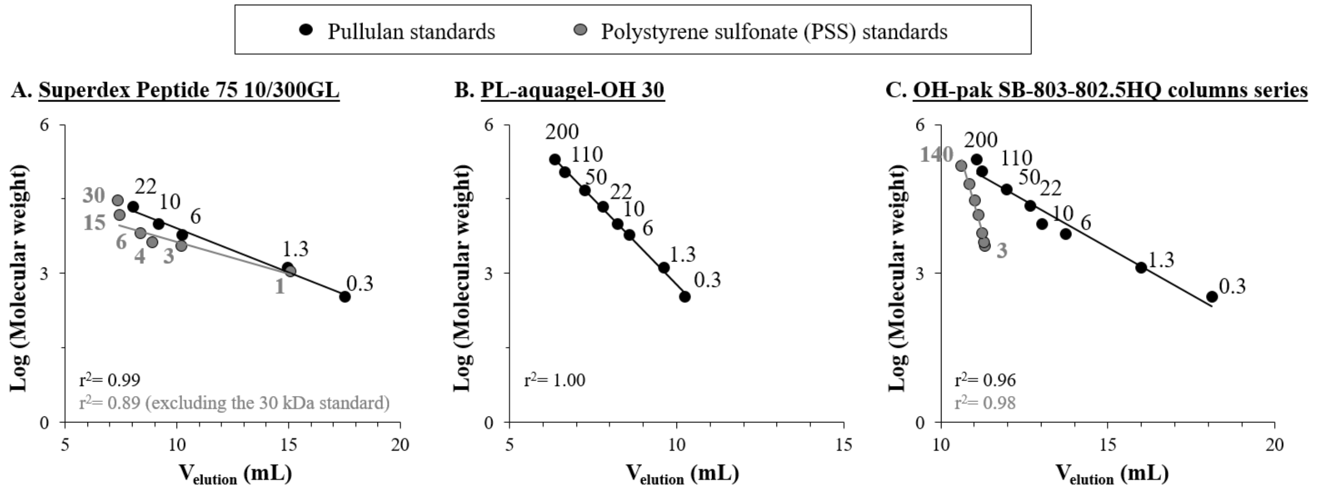

Supplementary Figure 2. Calibration in size of the three tested SEC columns using pullulan and polystyrene sulfonate (PSS) molecular weight standards. Panels A, B, and C show the size calibration obtained for respectively, the Superdex Peptide column, the PL-aquagel-OH 30 column, and the OH-pak SB-803-802.5HQ columns series. The mobile phase contained 5 mmol L^{-1} ammonium nitrate at pH 7 and the same results were obtained with 5 mmol L^{-1} ammonium nitrate at pH 9.5). On each plot, the number written aside each data point (above or below the trend line) represents the molecular weight, expressed in kDa, of each analyzed pullulan and PSS standard. For the Superdex peptide column (panel A), the PSS standards of 15 kDa and 30 kDa elute in the void volume and so the trend line is calculated excluding the 30 kDa PSS standard. For all other standards and columns, the trend lines are calculated considering all data points. The pullulan and PSS standards were detected as carbon and sulfur using the ICP-MS/MS.

The highest number of element-peaks were obtained with the OH-pak SB-803-802.5HQ columns series for both types of extracts, i.e., 11 peaks versus 7 peaks with the PL-aquagel OH30 column (Supplementary Figure 1). The OH-pak SB-803-802.5HQ columns series was also ideal for peak deconvolution because it provided, for Se and other elements, well-defined peaks with a better return to baseline between the peaks as compared to the PL-aquagel OH30 column. In addition, with the PL-aquagel OH30 column, the polystyrene sulfonated (PSS) standards did not elute out using 5 mmol L^{-1} NH_4NO_3 as a mobile phase (Supplementary Figure 2, panel B), indicating too strong hydrophobic interactions with this column that can lead to incomplete element recovery. We therefore selected the OH-pak SB-803-802.5HQ columns series.

b) **SEC separation of (trace) elements with different NH_4NO_3 concentrations in the mobile phase and selection of the optimal NH_4NO_3 concentration**

Up to 100 mmol L^{-1} salt concentration in the mobile phase is conventionally used to reduce ionic interactions between the analytes and the SEC stationary phase[e.g., 52,53]. In our case, the selected column is made of a hydroxylated polymethacrylate polymer that exhibits negative charges. Therefore, negatively charged Se species [e.g., SeO_3^-(IV) ion] or OM compounds can elute earlier than expected due to their repulsion. This is shown in panel C of Supplementary Figure 2, where the negatively charged PSS standards elute much earlier than the pullulan standards of same molecular weight. To reduce ionic interactions, we tested three NH_4NO_3 concentrations in the mobile phase for both NaOH and water extracts as illustrated in Supplementary Figure 3 with data obtained for the Kohala topsoil S6.

As expected, increasing NH_4NO_3 concentrations in the mobile phase increased the volume of elution of free SeO_3^{2-}(IV), from ~13.8 mL to ~17.2 mL (Supplementary Figure 3), showing the reduction of ionic repulsion of negative charge species when increasing salt concentrations. However, the increase in NH_4NO_3 concentrations also decreased the resolution of the separation of Se and of other elements (Supplementary Figure 3) as well as the column recovery of Se and other trace elements (Supplementary Table 4). For water and NaOH extracts respectively, up to 19 and 45% less Se eluted out of the column with 50 mmol L^{-1} NH_4NO_3 as compared to 5 mmol L^{-1} NH_4NO_3. These results can be explained by OM aggregation with increasing ionic strength as recently reported[53,58,59]. Therefore, 5 mmol L^{-1} NH_4NO_3 was selected as the optimal salt concentration in the mobile phase.

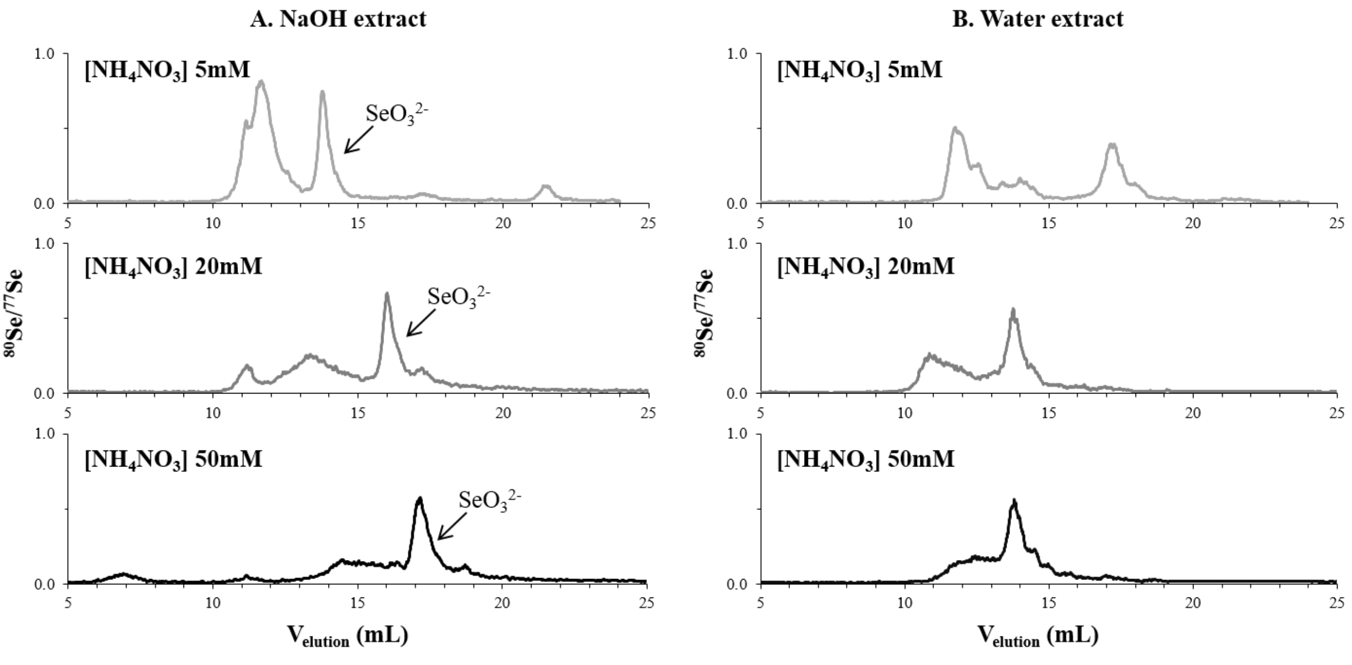

Supplementary Figure 3. Effect of ammonium nitrate (NH_4NO_3) concentrations in the mobile phase on the SEC separation of selenium (Se) in NaOH (panel A) and ultrapure water (panel B) extracts of a Kohala topsoil. The shown data are ^{80}Se chromatograms normalized by ^{77}Se added post-column (as internal standard) for the NaOH extracts (panel A) and ultrapure water extracts (panel B) of the topsoil from Kohala site S6 using the OH-pak SB-803-802.5HQ columns series and different NH_4NO_3 concentrations in the mobile phase (with methanol being fixed at 0%).

Supplementary Table 4. Effect of ammonium nitrate (NH_4NO_3) concentrations in the mobile phase on the amount of selenium (Se) eluting out of the optimal SEC column for NaOH and water extracts of Kohala topsoils from sites S1, S4, and S6. The methanol concentration of the mobile was fixed to 0%.

		5 mmol L^{-1}	20 mmol L^{-1}	50 mmol L^{-1}
**Extract type**	**Soil sample**	**Peak areas**	**Peak areas** *(average Se loss)*	**Peak areas** *(average Se loss)*
NaOH extract	S1	**11** ±1	6± 1 (*-45%*)	5 ± 1 (*-54%*)
	S4	**72** ± 7	51 ± 8(*-28 %*)	43 ± 10 (*-41%*)
	S6	**110** ± 11	95 ± 9(*-13%*)	78 ± 13 (*-29 %*)
Water extract	S1	**1.06** ± 0.05	1.0 ± 0.1 *(-4 %)*	0.86 ± 0.04 (*-19%*)
	S4	**3.9** ± 0.2	3.9 ± 0.2 (*-1%*)	3.5 ± 0.2 (*-10%*)
	S6	**7.5** ± 0.4	7.2 ± 0.4 (*-4%*)	6.8 ± 0.3 (-9%)

The results are presented as sum of ^{80}Se peak areas normalized by ^{77}Se (internal standard). The losses given in brackets were calculated with respect to the data obtained at 5 mmol L^{-1} NH_4NO_3. The given peak area average and standard deviation correspond to the injection in triplicate of each extract for each test of mobile phase ammonium nitrate concentrations.

c) SEC separation of (trace) elements with different MeOH concentrations in the mobile phase and selection of the optimal MeOH concentration

We tested the effect of methanol (MeOH) concentrations in the mobile phase on the SEC elution of Se (and other elements) in NaOH extracts (Supplementary Figure 4 for S6 topsoil), because MeOH is known to reduce hydrophobic interactions between the analytes and the SEC stationary phase[e.g., 53].

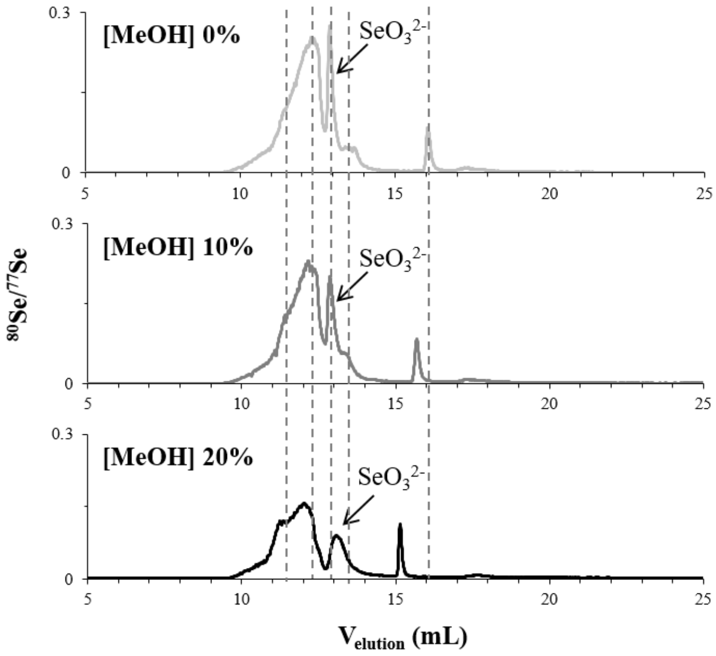

Supplementary Figure 4. Effect of methanol (MeOH) concentrations in the mobile phase on the SEC separation of selenium (Se) in NaOH extract of a Kohala topsoil. The shown data are ^{80}Se chromatograms normalized by ^{77}Se added post-column (internal standard) for the NaOH extract of the topsoil from Kohala site S6 using the OH-pak SB-803-802.5HQ columns series and different MeOH concentrations in the mobile phase (with ammonium nitrate concentration fixed at 5 mmol L^{-1}).

When increasing MeOH concentrations in the mobile phase, i.e., when using 20% MeOH versus no MeOH, the SEC-peaks of Se (and other TEs) eluted slightly earlier, i.e., by ~0.4 mL for the first two Se-peaks and up to ~0.9 mL for the last Se-peak. Therefore, there were hydrophobic interactions of the Se species with the stationary phase of the selected column. However, these interactions did not modify the elution profile of Se, with the five same Se SEC-peaks being detected with 0 and 10% MeOH. With 20% MeOH, all Se-peaks were wider, including the peak of free SeO_3^{2-}(IV) that was consequently co-eluting with the fourth Se-peak. Because increasing MeOH concentrations in the mobile phase did not have a significant effect on Se elution but broadened the Se-peaks and decreased ICP-MS/MS sensibility (due to the requirement of adding Ar/O_2 gases mixture in the plasma), 5 mmol L^{-1} NH_4NO_3 without MeOH was selected as the optimal mobile phase. In addition, a mobile phase without MeOH provides the possibility to measure C together with Se and other elements.

Supplementary Discussion 3. Classification of elements into fractions of different size and chemical properties

a) Targeted size and size determination with the optimized SEC-UV-ICP-MS/MS method

Targeted size range. Our selected optimal SEC column separates (macro)molecules that have sizes ranging between 0.3-100 kDa and nanoparticles of size <~40 nm, based on the columns pore size communicated by the manufacturer. The selected SEC size range thus targets a separation of free Se oxyanions, organic Se fractions of different size and chemical properties, and of small (organo)mineral nanoparticles. It is important to note that the SEC-UV-ICP-MS/MS method (that is applicable to soil extracts) does not reflect the complete particle composition of soils, as most methods used to estimate (e.g., sequential extractions and total element quantification or hydride generation-AFS) or to determine (e.g., LC-ICP-MS) soil Se speciation. This is due to the soil sample preparation step (soil sieving to remove particles >2 µm because trace elements are enriched in the clay (<2µm) and silt (2-50 µm) fractions) as well as the extraction step (followed by centrifugation and filtration of the soil extracts at max. 0.45 µm, needed before injection into analytical instruments). The common filtration of the extracts at 0.45 µm and the size separation of our optimized SEC (0.3-100 kDa and <~40 nm, which is the column pore size) do also not entirely reflect the particle composition of soil extrcts. Indeed, the few previous studies investigating this reported that water extracts can contain particles of size up to few µm[60,61] and that alkaline extracts contain particles of size between 10 and 150 nm[62,63]. Although the size separation of our optimized SEC method does not cover the complete particle composition of NaOH extracts, all Se (and S) present in NaOH extracts was recovered i.e., the SEC Se species recoveries were 96-112 (101±5) % of total Se in NaOH extracts (n=25 soils; cf. Table 1 in manuscript and associated discussion). Therefore, there is no need for a method with a wider size range to determine Se speciation in alkaline extracts. In water extracts, we could recover 58-109 (81±15) % of total Se in water extracts (n=25 soils) with our optimal SEC method, and by quantifying elements in water extracts after filtration at 20 nm, we could observe that the unrecovered Se species by SEC are too big to elute out of the column (cf. Table 2 in manuscript and associated discussion). Therefore, a SEC column specific for larger nanoparticles or field flow fractionation is needed to identify and study the unrecovered fraction of Se in water extracts.

Size determination of SEC fractions. Despite thorough optimizations of the SEC separation, obtaining the exact size of the detected Se SEC-fractions using molecular weight standards remains difficult (as expected[64,58,53]), mainly due to ionic interaction (negative repulsion) between the analytes and the stationary phase of the selected Shodex OH-PakSB803-802.5 columns, which are negatively charged. Indeed, there were important shifts toward higher retention time for the SEC-peaks of soil extracts when increasing ammonium nitrate concentrations (Supplementary Figure 3). In addition, all the PSS molecular weight standards, which are negatively charged, elute much earlier than the pullulan standards, which are neutral compounds (Supplementary Figure 2). There was, however, only a small shift in retention time for the SEC-peaks of soil extracts when increasing MeOH concentration of the mobile phase (Supplementary Figure 4), meaning that hydrophobic interactions do not affect the separation of analytes by size. Despite the ionic interactions occurring during our SEC separation, many isolated and purified humic acids eluted earlier with our optimized SEC than fulvic acids (Supplementary Figure 5), which is consistent with the higher average molecular weight of humic versus fulvic acids, and so the organic matter (OM) is separated by size with our optimized SEC method.

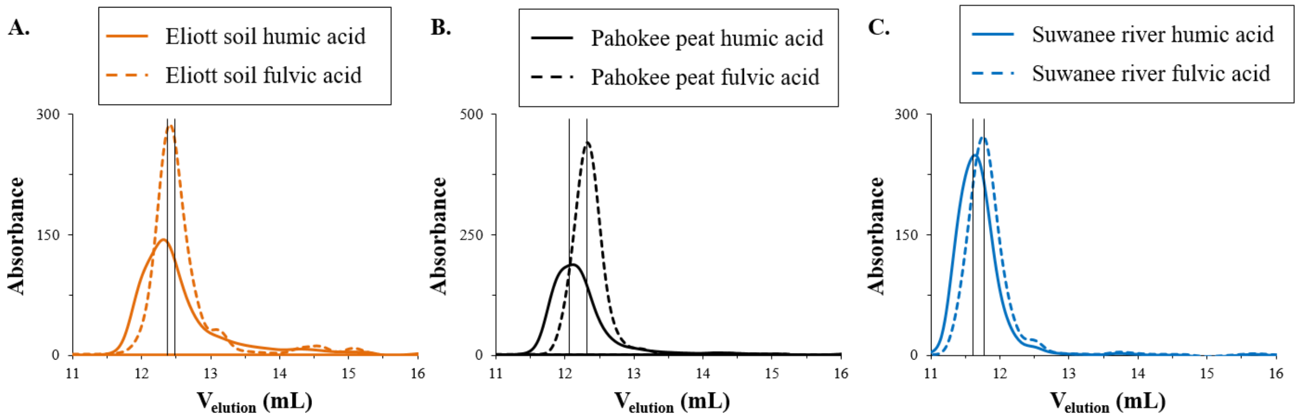

Supplementary Figure 5. Chromatograms of ultraviolet (UV) absorbance obtained with the optimized SEC separation for isolated and purified humic and fulvic acid materials from the International Humic Substances Society. Panel A shows the UV chromatograms obtained for the humic and fulvic acids from Eliott soil. Panel B shows the UV chromatograms obtained for the humic and fulvic acids from Pahokee peat. Panel C shows the UV chromatograms obtained for the humic and fulvic acids from Suwanee river. The plain vertical black line show the apex of the UV peak(s).

b) Fraction F1: (Organo)mineral nanoparticles

F1 was defined by the elution of large amounts of iron (Fe) and titanium (Ti), i.e., two peaks in NaOH, apex at ~11.2 and 11.4 mL and one peak in water, apex at ~11.0 mL to which some arsenic (As) and most of the lead (Pb) are associated (Supplementary Figure 6). F1 thus consisted of (oxyhydr)oxides nanoparticles, mobilized from soils (water and NaOH extracts) and potentially formed during NaOH extractions (due to high pH), to which As and Pb have high affinity[62,65]. In agreement with our finding, Neubauer et al.[62] showed by asymmetrical field flow fractionation that most Fe exist as ~10-30 nm (oxy)hydroxide colloids (to which Pb binds to) in soil alkaline and water extracts. In our study, there were no carbon (C) and UV peaks co-eluting with these Fe-, Ti-, Pb- and As-peaks in NaOH extracts of S1 to S4 topsoils containing 1 to 9% of SOC. In contrast, for S5 and S6 topsoil NaOH extracts and for all water extracts (Supplementary Figure 6), there was an UV peak or a peak shoulder to the main UV peak co-eluting with these Fe-, Ti-, Pb- and As- peaks (but no C-peak). This UV feature can result from

the presence of Fe(III) or other metal species (e.g., manganese (Mn) or aluminum (Al)) which absorb UV light[66] (probably the case in NaOH extracts), and/or from associations of OM to mineral colloids (probably in water extracts).

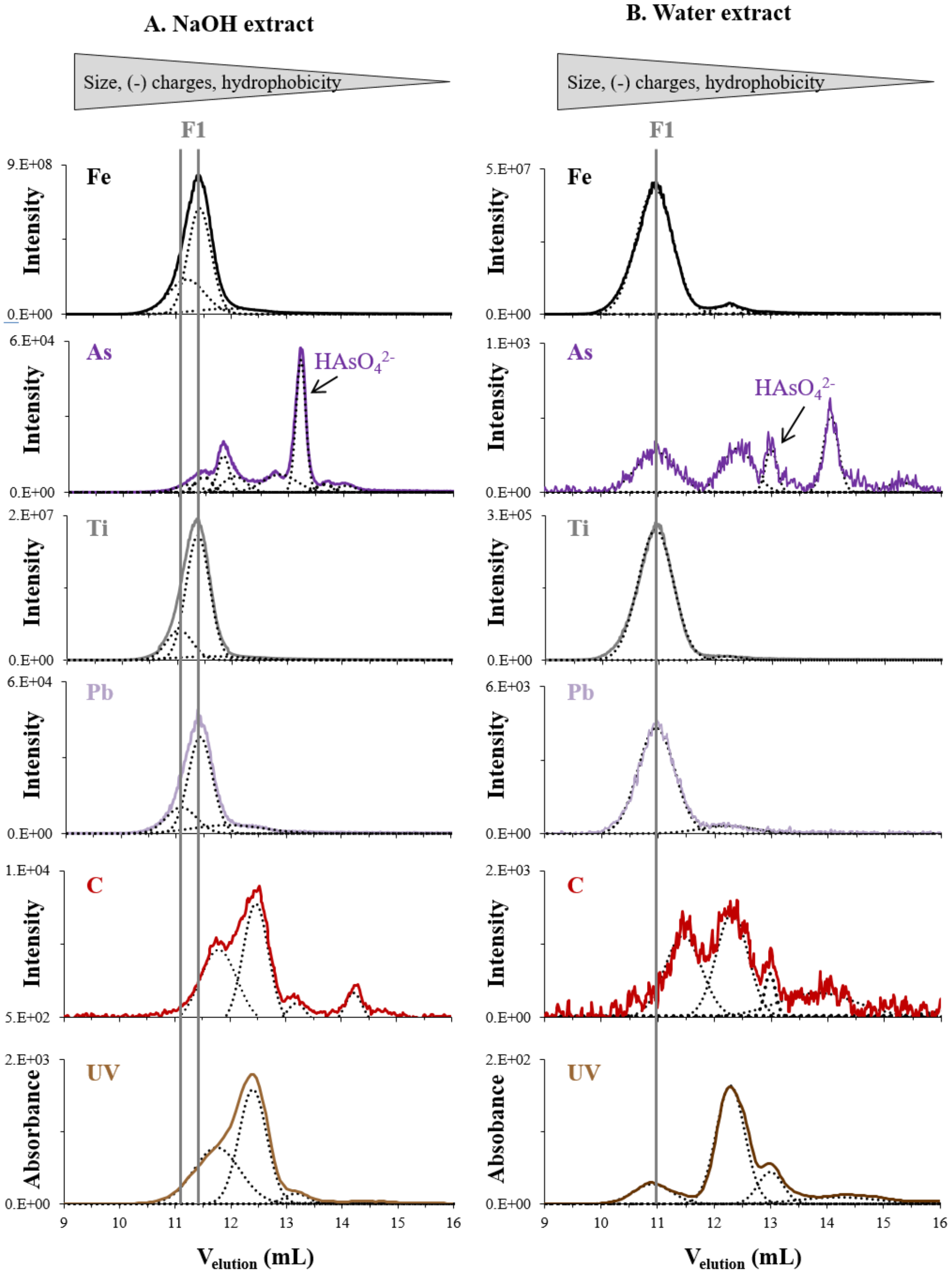

Supplementary Figure 6. Identification of SEC fraction F1 in soil extracts based on co-elution of titanium (Ti) and lead (Pb) with iron (Fe) and arsenic (As), which is illustrated with the chromatograms obtained for the extracts of the topsoil from Kohala site S4 (containing medium SOC concentration). Panel A shows the Ti, Pb, Fe and As chromatograms obtained for the NaOH extract, while panel B shows the Ti, Pb, Fe and As chromatograms obtained for the ultrapure extract. The dashed lines show the deconvoluted peaks assigned to fraction F1, and the vertical lines show the peak apex. Two peaks containing e.g., Fe, As, Ti are defining fraction F1 in NaOH extracts and one peak containing .g., Fe, As, Ti is defining fraction F1 in water extracts.

c) Fractions F2 and F3: larger, more hydrophobic and/or negatively charged, OM fractions

F2 and F3 were primarily defined from features in the C and UV signal supported by other elements (Fe, sulfur (S) and copper (Cu)) and by the elution of reference Eliott soil humic and fulvic acids (Supplementary Figure 7). In NaOH extracts, F2a included three peaks (Supplementary Figure 7, panel A): i) the first C- and UV-peak that was associated with Cu, Se and As (apex, 11.8 mL); ii) the S-peak eluting slightly earlier (apex, 11.6 mL); and iii) the Fe- and As-peak eluting slightly later (apex, 12.0 mL). F3 included the 2nd and 3rd C- and UV-peaks (apex, ~12.4 and 13.2 mL) associated with Cu, Se and S.

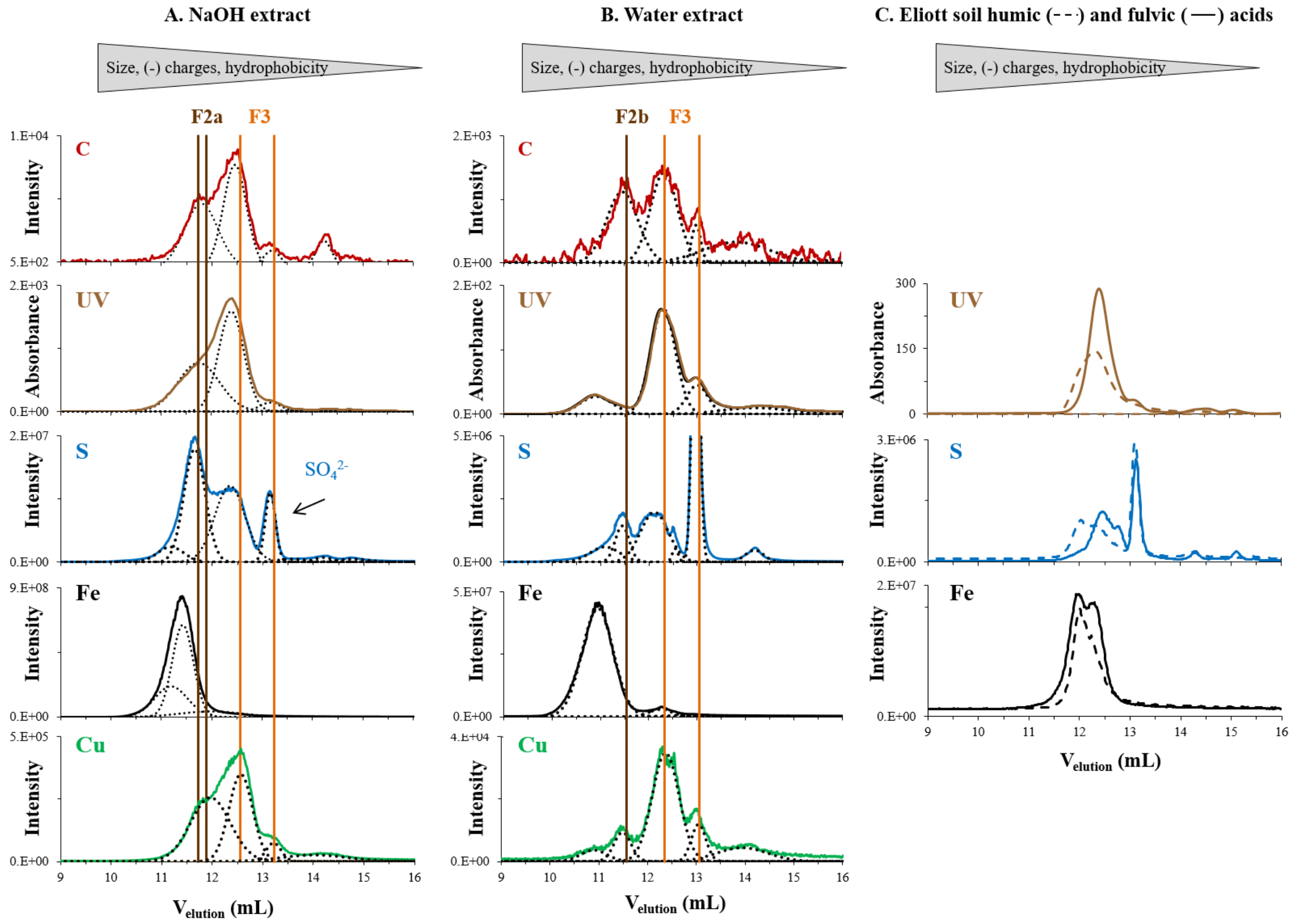

Supplementary Figure 7. Identification of SEC fractions F2 and F3 in NaOH and ultrapure water extracts based on (co)elution of carbon (C), UV, copper (Cu), sulfur (S), iron (Fe) and/or arsenic (As). The panels A and B show the UV and elemental SEC chromatograms obtained for the NaOH (panel A) and water extract (panel B) of the topsoil from Kohala sites S4 (that contain medium SOC concentration). The panel C shows the UV and elemental elution of reference Eliott soil humic and fulvic acids. In panels A and B, the dashed lines show the deconvoluted peaks assigned to fraction F2a, F2b and F3, and the vertical lines show the apex of these peaks.

The SEC elution of UV, Fe and S of the fractions F2a and F3 defined for our NaOH soil extracts matched very well the UV, Fe and S elution of Eliott soil humic and fulvic acids, respectively. The UV signal of Eliott soil humic acid (apex, ~11.6 mL) elutes slightly before the one of Eliott soil fulvic acid, which is composed of 2 main UV-peaks (apex, ~11.8 and 12.6 mL; Supplementary Figure 7, panel C). Similarly, the fraction we defined as F2 elutes slightly before the fraction we defined as F3, which is composed of

two main UV-peaks (Supplementary Figure 7, panel A). In addition, the UV signal of Eliott soil humic acid has lower UV intensities but is wider than the main UV-peak of Eliott soil fulvic acid, which is also the case for the fraction F2 of our NaOH extracts with respect to the fraction F3. The different elution of humic and fulvic acids is explained by more interactions occurring between the molecules composing humic acids, thus forming molecular structures of more variable size but that are overall bigger than the fulvic acids[64,67]. Another similarity between F2a and Eliott soil humic acid is the S-peak eluting ~0.2 mL slightly earlier than the UV-peak, suggesting that S is enriched in the bigger and/or more hydrophobic humic acid fraction. Finally, in agreement with the Fe-peak being assigned to F2a (and not F3), the Fe associated to Eliott soil fulvic acid elutes earlier than its UV signal and rather matches the UV-peak apex of Eliott soil humic acid. In agreement with the Fe elution we obtain for our NaOH extract and references humic substances, isolated humic acid was shown to have an higher Fe complexation capacity and to form more stable complexes as compared to FA materials[68,69]. Overall, in NaOH extracts, F2a represents larger, more hydrophobic and/or negatively charged, Fe-enriched, aromatic OM whereas F3 represents smaller, less hydrophobic and/or negatively charged, aromatic OM that contains no or less Fe.

In water extracts, there was no UV signal associated to the first C-peak (apex, ~11.5 mL; Supplementary Figure 7, panel B) as for the NaOH extracts. Therefore, we defined F2b as larger and aliphatic, non-Fe enriched OM, in contrast to fraction F2a of NaOH extracts that corresponds to larger, more hydrophobic and negatively charged, Fe-enriched, aromatic OM. The absence of bigger and/or more hydrophobic aromatic, Fe-containing, OM (more humic acids-type) and presence of an aliphatic OM fraction in water extracts is not surprising, considering that previous studies showed that humic acids do not account for much of soil DOM, instead up to 40% of soil DOM can be aliphatic[70,71]. In water extracts, the second and third C-peaks (apex, ~11.2 and 13 mL; defined as F3) were associated to strong UV signals as observed in NaOH extracts. Therefore, F3 represents smaller, less hydrophobic and/or negatively charged, aromatic OM in water extracts as in NaOH extracts (Supplementary Figure 7, panel A-B). F3 was associated to clear Cu-, S-, Se-, Fe- and As-peaks, while F2b was associated to Cu- and S-peaks but not to Fe- and As-peak, in line with previous studies suggesting an higher affinity of Fe(III) for aromatic OM compounds[68].

d) Fractions F4 and F5: Small hydrophilic compounds and free oxyanions

F4 encompassed all other elemental peaks eluting after ~13.6 mL (see zoom on Supplementary Figure 8, panels A and B), which had very low or no UV peak. F4 consists of small hydrophilic organic compounds.

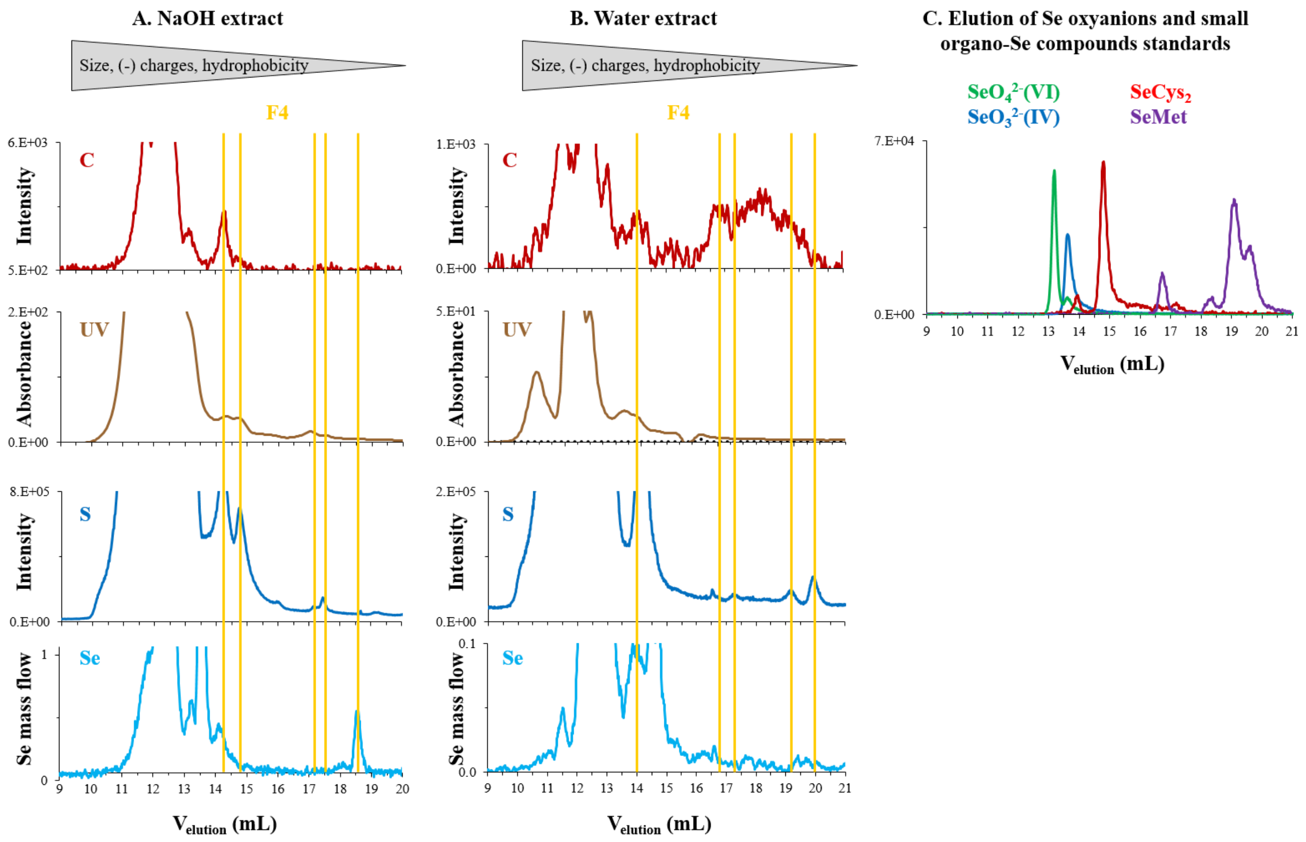

Supplementary Figure 8. Identification of SEC fractions F4 and F5 in NaOH and ultrapure water extracts based on elution of carbon (C), UV, sulfur (S) and selenium (Se) as well as on elution of Se standards. The panels A and B show a zoom on the UV and elemental chromatograms obtained for the NaOH (panel A) and water (panel B) extracts of the topsoil from Kohala site S4 (that contains medium SOC concentration), which highlight the small peaks eluting after 13.7 mL and corresponding to the fraction F4 Small hydrophilic OM. The panel C shows the SEC chromatogram of SeO_4^{2-}(VI), SeO_3^{2-}(IV), $SeCys_2$ and SeMet standards (prepared in ultrapure water at 10 µg L^{-1}).

High molecular weight neutral compounds that would elute later because of hydrophobic interactions with stationary phase of the column can be discarded because when adding MeOH in the mobile phase these elemental peaks still elute after ~13.7 mL; Supplementary Figure 4 in Supplementary Discussion 4). In agreement with our interpretation, small hydrophilic organic compounds not absorbing UV-light[53,72] have previously been shown to elute toward the end of SEC. F4 may include small Se metabolites from plants and/or bacteria, but other than SeMet and $SeCys_2$. Indeed, although these two compounds eluted within the elution volume range of fraction F4 (Supplementary Figure 8), they did not match the SEC-peaks observed after 13.6 mL for the NaOH and water soil extracts. In addition, SeMet and $SeCys_2$ were not detected in soil extracts with AEC-ICP-MS/MS. Finally, F5 corresponded to the Se, S and As free ions (i.e., SeO_3^{2-}, SO_4^{2-} and $HAsO_4^{-}$), whose elution volumes were confirmed by standard additions to the extracts.

Supplementary Discussion 4. Comparison between Se oxyanions quantified by AEC-ICP-MS/MS and SEC-UV-ICP-MS/MS

A very good agreement was obtained between the concentrations of free SeO_3^{2-}(IV) obtained in NaOH extracts by SEC-UV-ICP-MS/MS and by AEC-ICP-MS/MS ($[Se(IV)]_{SEC}=0.91x[Se(IV)]_{AEC}$, $r^2=0.93$,

p<0.01; n=25 soils; Supplementary Figure 9, panel A). Note that no SeO_4^{2-}(VI) was detected in NaOH extracts. Similarly, a good agreement was obtained between the concentrations of free Se oxyanions (sum of SeO_3^{2-}(IV) and SeO_4^{2-}(VI)) obtained in the water extracts by SEC-UV-ICP-MS/MS and by AEC-ICP-MS/MS (($[Se(IV)+Se(VI)]_{SEC}$=1.12x$[Se(IV)+Se(VI)]_{AEC}$, r=0.84, p<0.01; n=16 soils because no Se oxyanion was detected in soils from sites S5 and S6; Supplementary Figure 10, panel B).

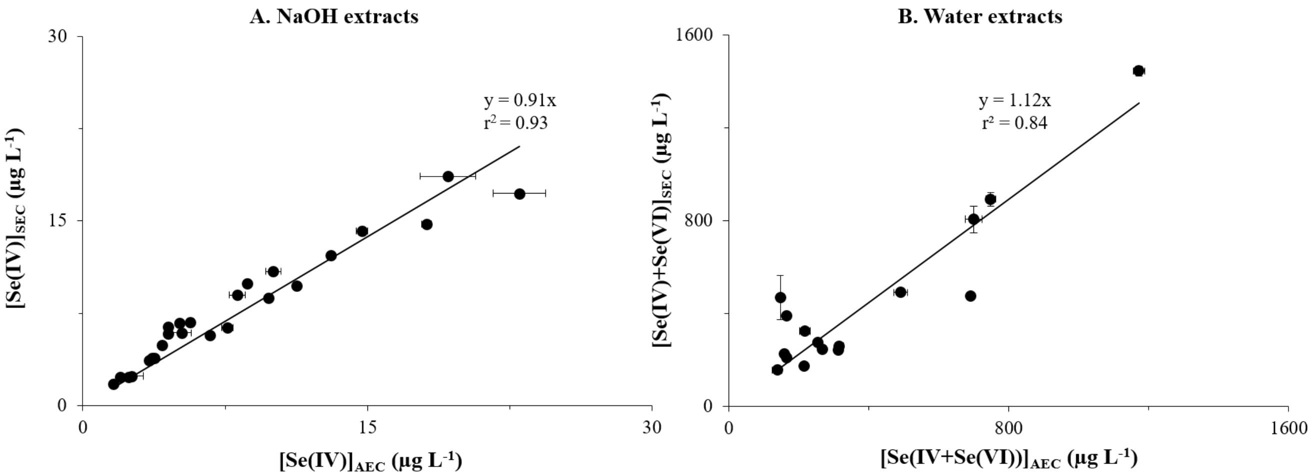

Supplementary Figure 9. Good agreement between the concentrations of free Se oxyanions (SeO_3^{2-}(IV)+SeO_4^{2-}(VI)) obtained in soil extracts by SEC-UV-ICP-MS/MS and by AEC-ICP-MS/MS. The panel A shows the 2-tailed Spearman correlation (p<0.001) between free SeO_3^{2-}(IV) concentrations obtained by SEC-UV-ICP-MS/MS ($[Se(IV)]_{SEC}$; y-axis) and AEC-ICP-MS/MS ($[Se(IV)]_{AEC}$; x-axis) for the NaOH extracts of the 25 analyzed soils (no Se(VI) was present in NaOH extracts). The panel B shows the 2-tailed Spearman correlation (p<0.001) between free Se oxyanions concentrations obtained by SEC-UV-ICP-MS/MS ($[Se(IV)+Se(VI)]_{SEC}$; y-axis) and AEC-ICP-MS/MS ($[Se(IV)+Se(VI)]_{AEC}$; x-axis) for the water extracts of the 16 analyzed soils where Se oxyanions were detected. The error bars for the y variables, i.e., $[Se(IV)]_{SEC}$ in panel A and $[Se(IV)+Se(VI)]_{SEC}$ in panel B, represent the standard deviations associated to the deconvolution of the SEC peak corresponding to Se(IV)+Se(VI). The error bars for the x variables, i.e., $[Se(IV)]_{AEC}$ in panel A and $[Se(IV)+Se(VI)]_{AEC}$ in panel B represent the standard deviations associated to the injection of each extract in duplicate during the AEC-ICP-MS/MS analysis. Note that for the parameter $[Se(IV)+Se(VI)]_{AEC}$, the shown standard deviation includes the propagation of the error associated to the sum up of the concentrations of Se(IV) and Se(VI) obtained in duplicate.

Supplementary Discussion 5. Stability of the SEC-ICP-MS/MS analysis during long runs

The extracts from the 25 studied Kohala soils were measured in separated sessions at different dates, i.e., one for the NaOH extracts and one for the water extracts. Supplementary Figure 10 shows the between-sample variability of scandium (^{45}Sc) and yttrium (^{89}Y) internal standards (Supplementary Table 3) during the SEC-UV-ICP-MS/MS analysis of all Kohala soil extracts, which lasted for ~30 consecutive hours for the NaOH extracts and ~60 hours (with a small break in the batch) for the water extracts. The between-sample variability ($V_{between\ sample}$) of each internal standard, expressed in %, was calculated as shown in equation (1).

$$V_{between\ sample} = \frac{I_{t=1}}{I_{t=h}} \times 100 \qquad (1)$$

with $V_{between\ sample}$ being the between-sample variability of the internal standard, $I_{t=1}$ representing the average intensity of the internal standard throughout the chromatogram of the first analyzed sample and $I_{t=h}$ representing the average intensity of the internal standard throughout the chromatogram of the following analyzed samples with h going up to 30 hours (for NaOH extracts) and 60 hours (for water extracts).

For both runs, 6 of the 25 analyzed samples were analyzed in triplicate together with blanks, standards of Se species (Se(IV), Se(VI), SeMet, $SeCys_2$), As species (As(III) and As(V)) and S species (S(VI)), molecular weight standards (pullulan and polystyrene sulfonated standards) as well as soil extracts spiked with Se(IV) and Se(VI). The run of the water extracts was longer because some measurements were reported due to a non-optimal concentrations of post-column added ^{78}Se(IV) used to quantify Se in SEC peaks by on-line isotope dilution.

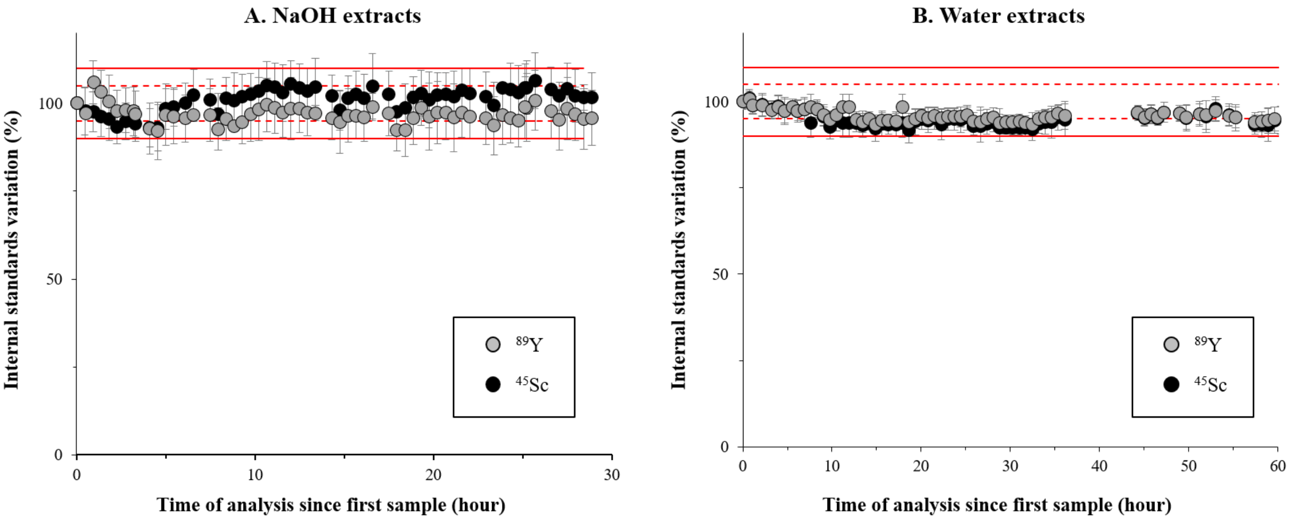

Supplementary Figure 10. Between-sample variability of scandium (^{45}Sc) and yttrium (^{89}Y) internal standards during the SEC-UV-ICP-MS/MS analysis of all Kohala soil extracts using H_2 in the collision/reaction cell. Panel A shows the ^{45}Sc and ^{89}Y variability during the analysis of NaOH extracts, while panel B shows the ^{45}Sc and ^{89}Y variability during the analysis of ultrapure water extracts. Information on internal standard addition and ICP-MS/MS detection are given in Supplementary Table 3. The between-sample variability of the internal standards is calculated following equation (1). The solid and dashed red lines show the boundaries for an internal standard variability of ±7% and ±5 %, respectively. Note that the error bar for each point (each point corresponding to an analyzed sample) represents the standard deviation associated to the internal standard variability within the SEC separation of the sample.

Supplementary Figure 10 shows that the variation of both ^{45}Sc and ^{89}Y internal standards was within ±7% for NaOH extracts and within ±5% for water extracts, meaning that the sensitivity of ICP-MS/MS with our SEC-UV-ICP-MS/MS is stable during long runs. More precisely, the averages of the ^{45}Sc and ^{89}Y variability over each entire runs were as follows: 101 ± 3% for ^{45}Sc and 97 ± 2% for ^{89}Y when analyzing the NaOH extracts, and 92 ± 2% for ^{45}Sc and 96 ± 2% for ^{89}Y when analyzing the water extracts.

Supplementary Discussion 6. Reproducibility of the SEC-UV-ICP-MS/MS method for Se speciation

The reproducibility of the SEC-UV-ICP-MS/MS analysis, including the Se quantification by on-line ID and peak deconvolution, was assessed through analyzing the water and NaOH extracts of the six Kohala topsoils in triplicate. The triplicates were distributed randomly within the water or NaOH extract runs. The relative standard deviations (RSD) of the Se SEC-peaks elution volume (synonym to retention time) were excellent, i.e., 0.3±0.4% and 0.2±0.3% for water and NaOH extracts, respectively. The reproducibility of the Se quantification in the SEC-fractions was reasonably good, i.e., on average 12±10% (range, 0.05-35 %) and 12±7% (range, 0.7-23 %) for water and NaOH extracts, respectively. The variability in Se concentrations in SEC-peaks was mostly driven by the peak deconvolution step rather than by the data acquisition itself as SEC chromatograms were highly reproducible (Supplementary Figure 11).

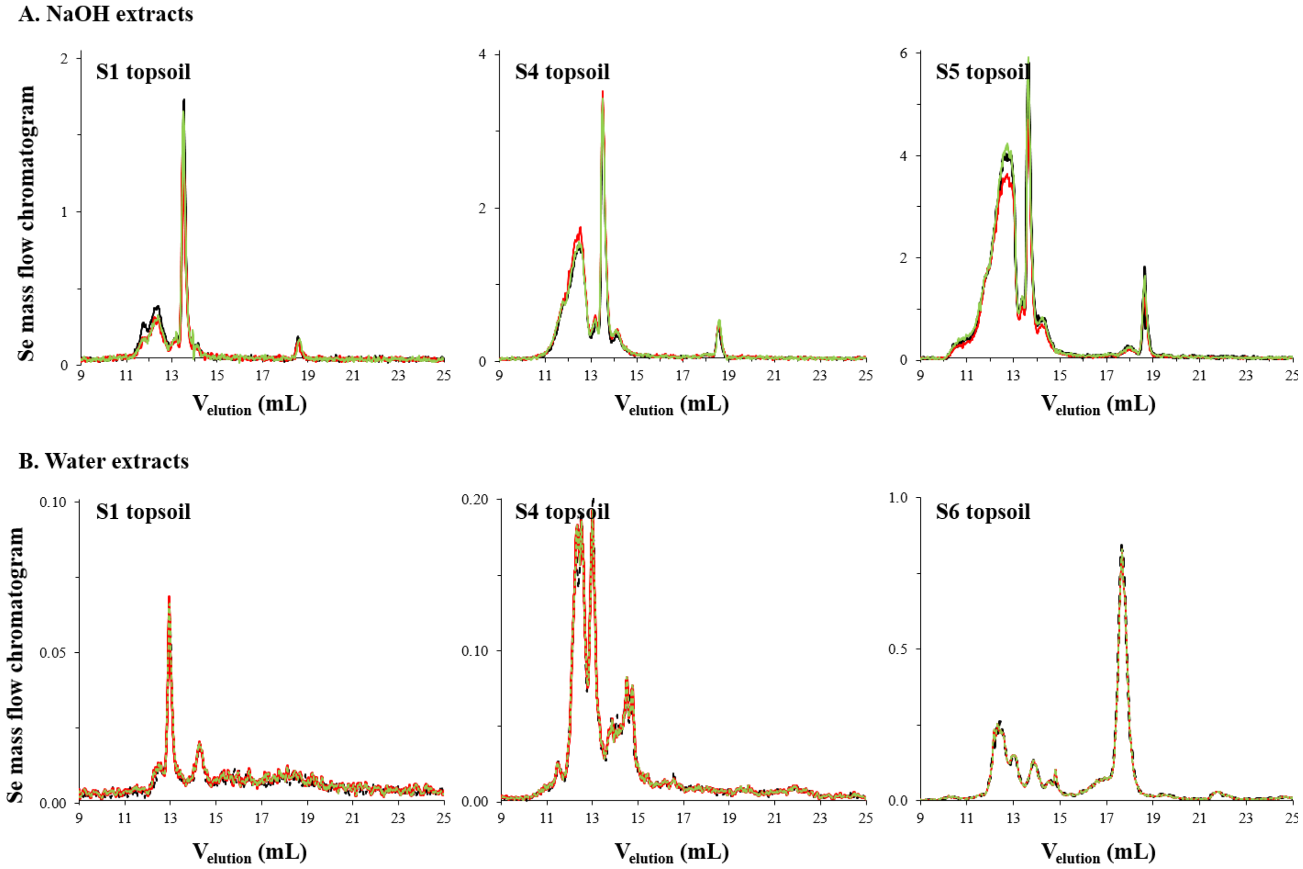

Supplementary Figure 11. Overlaid triplicate of selenium (Se) mass flow chromatograms obtained by SEC-UV-ICP-MS/MS for NaOH (panel A) and ultrapure water (panel B) extracts of Kohala topsoils (0-10 cm) from sites S1, S4, and S6.

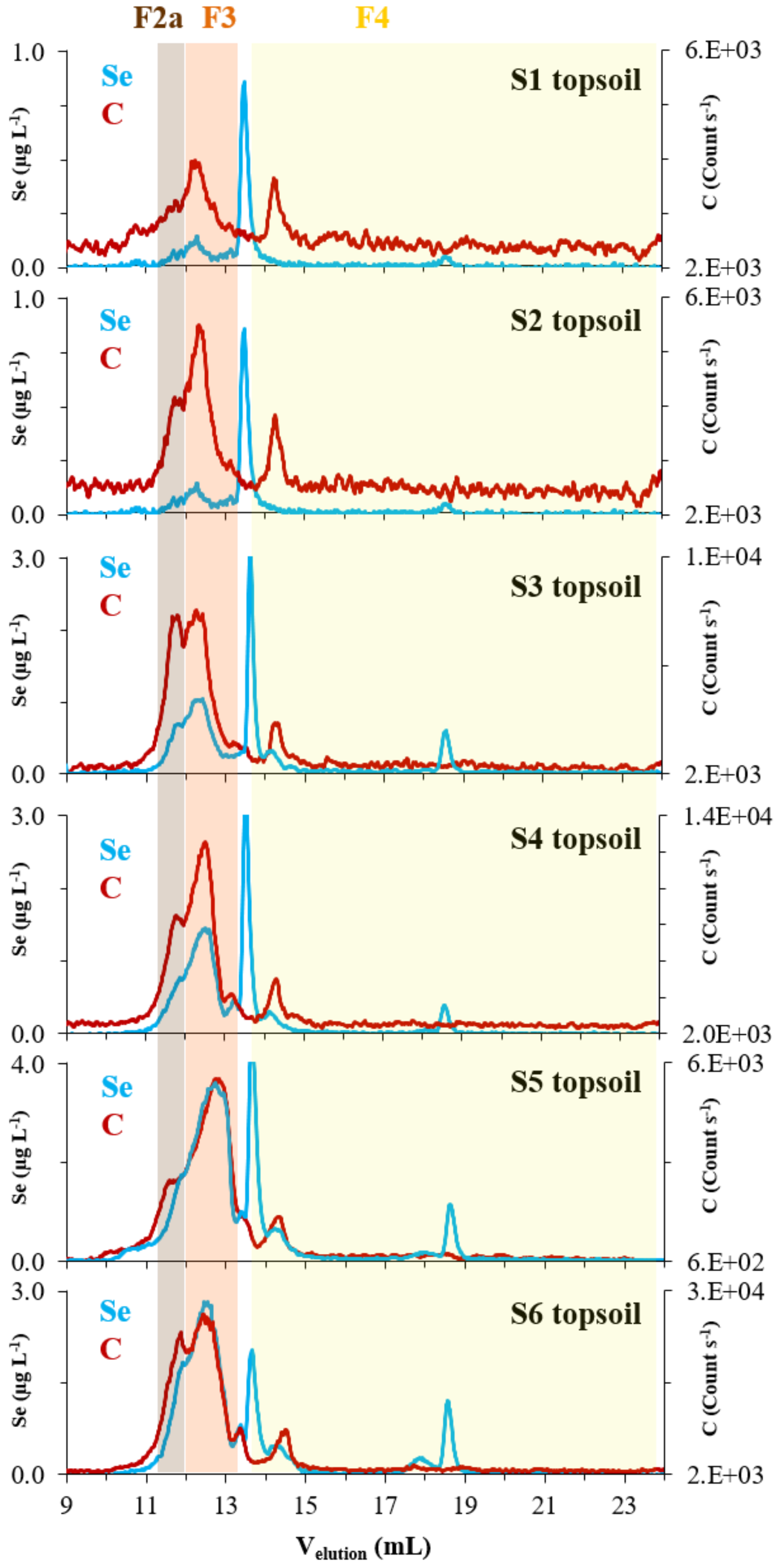

Supplementary Figure 12. Selenium (Se) and carbon (C) chromatograms for the NaOH extracts of the six Kohala topsoils (0-10cm) showing the concomitant decrease in the proportions of Se and C in the organic fractions F4 with respect to F2-F3 along the rainfall and SOC gradient. The vertical dashed lines show the apex of the identified F2a, F3 and F4 fraction peak(s).

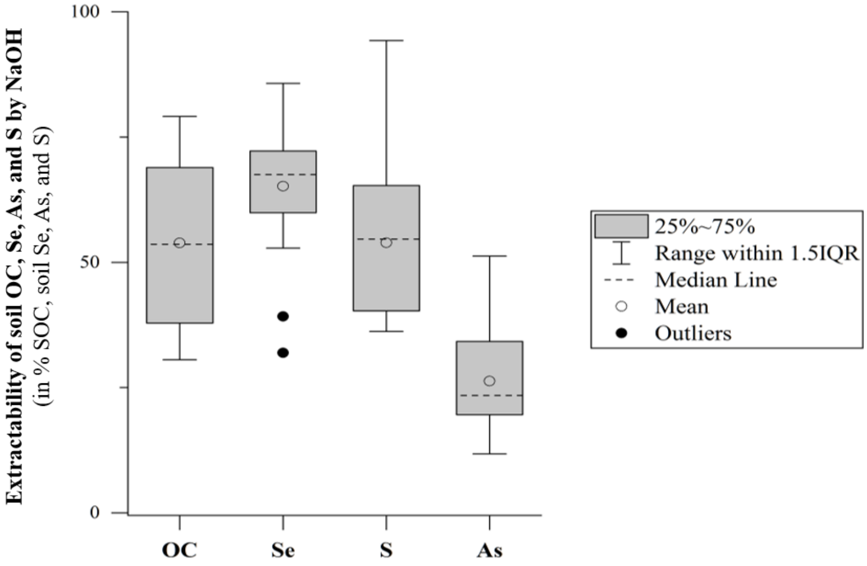

Supplementary Figure 13. NaOH extraction efficiency of organic carbon (OC), selenium (Se), sulfur (S) and arsenic (As) for all analyzed Kohala soils (n=25). The efficiencies are expressed in % of the total concentration of soil organic carbon (SOC), soil Se, S, and As.

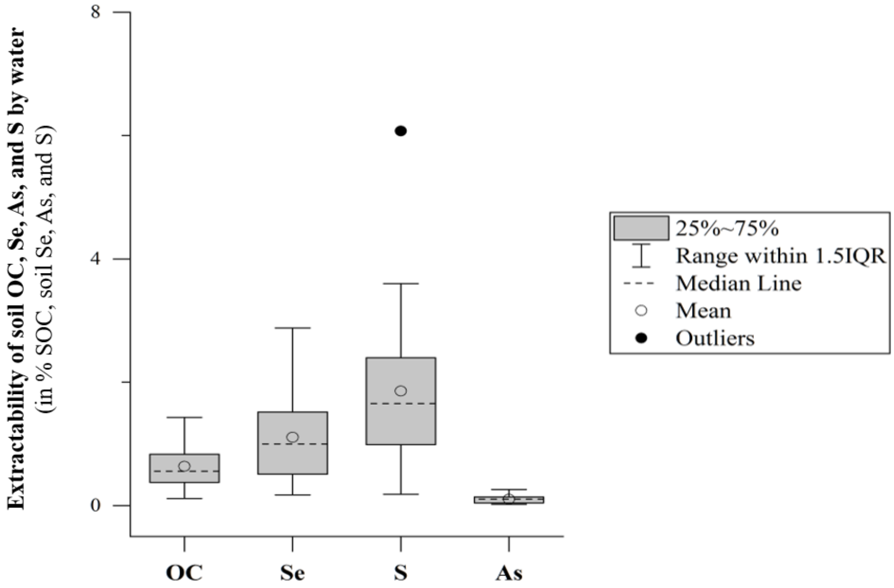

Supplementary Figure 14. Ultrapure water extraction efficiency of organic carbon (OC), selenium (Se), sulfur (S) and arsenic (As) for all analyzed Kohala soils (n=25). The efficiencies are expressed in % of the total concentration of soil organic carbon (SOC), soil Se, S, and As.

Supplementary Table 5. Dominant plant species at the six study Kohala sites for which leaves were analyzed.

**S1**	Buffel grass (*Pennisetum ciliare*)
**S2**	Buffel grass (*Pennisetum ciliare*)
**S3**	Kikuyu grass (*Pennisetum clandestinum*), and perennial soybean (*Neonotonia wightii*)
**S4**	Kikuyu grass (*Pennisetum clandestinum*) and perennial soybean (*Neonotonia wightii*)
**S5**	Kikuyu grass (*Pennisetum clandestinum*)
**S6**	Kikuyu grass (*Pennisetum clandestinum*) and ohia trees (*Metrosideros polymorpha*)

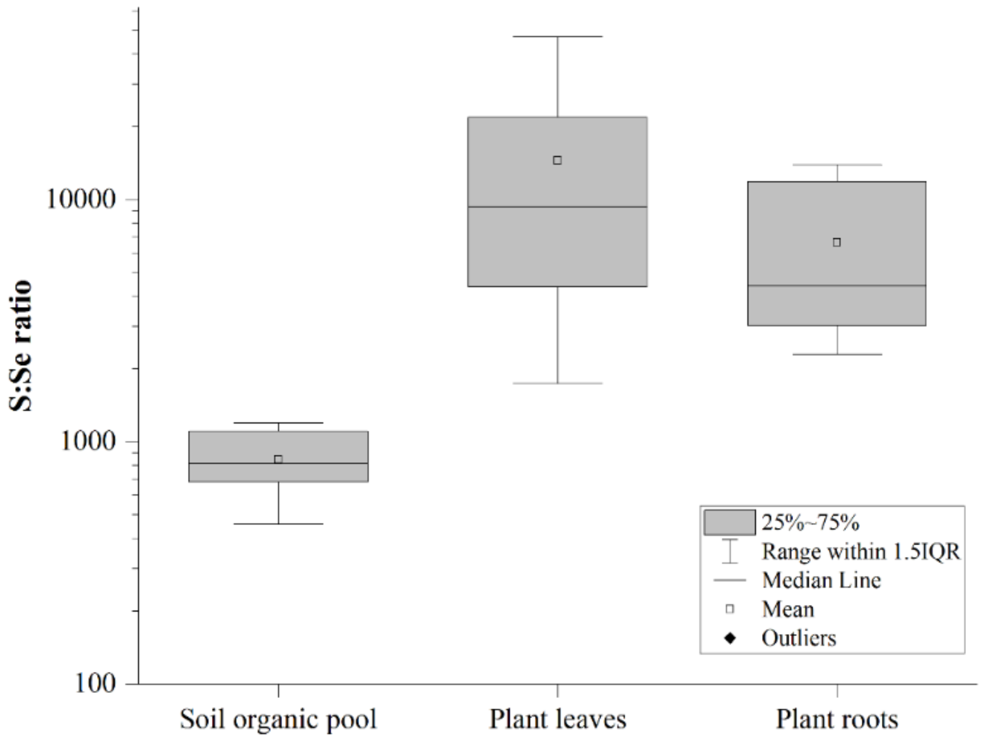

Supplementary Figure 15. Organic sulfur (S) to organic selenium (Se) ratio in all analyzed Kohala soils (n=25) and S to Se ratios in analyzed plant leaves (from grass, soybean and tree; n=9) and mixed roots (n=6).

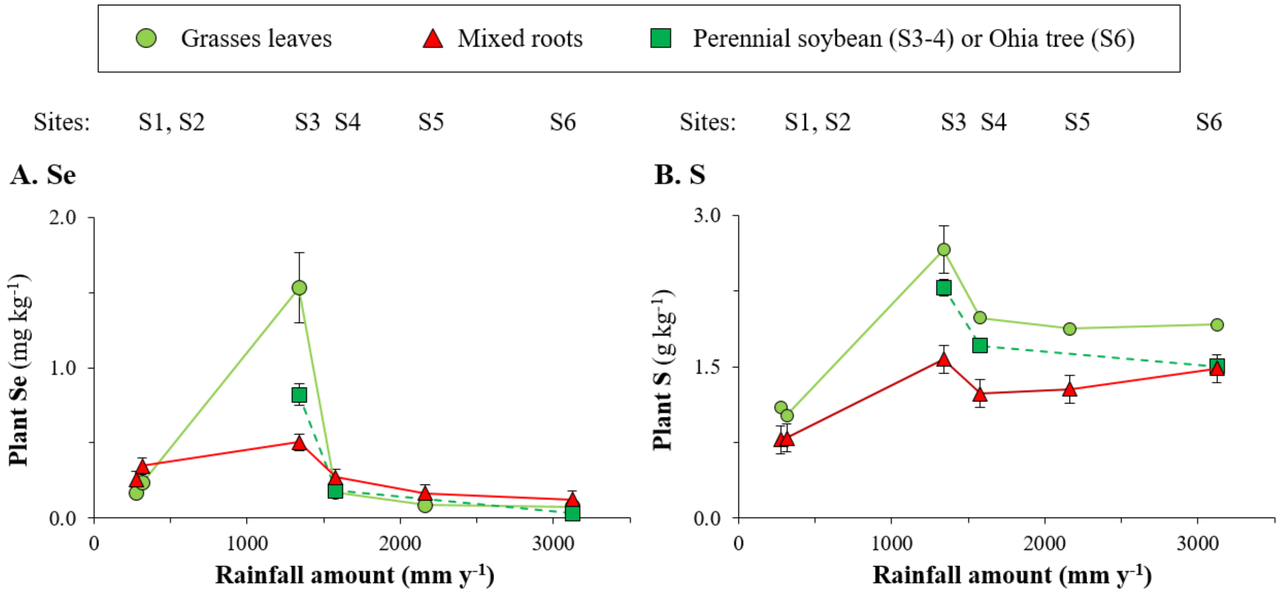

Supplementary Figure 16. Concentrations of selenium (Se, panel A) and sulfur (S, panel B) in plant leaves and mixed roots along the Kohala rainfall gradient. The error bars represent the standard deviation values resulting from the digestion of each Kohala plant material in triplicate and subsequent element quantification in each digestion triplicate. Information on the plant materials is given in Supplementary Table 5 above.

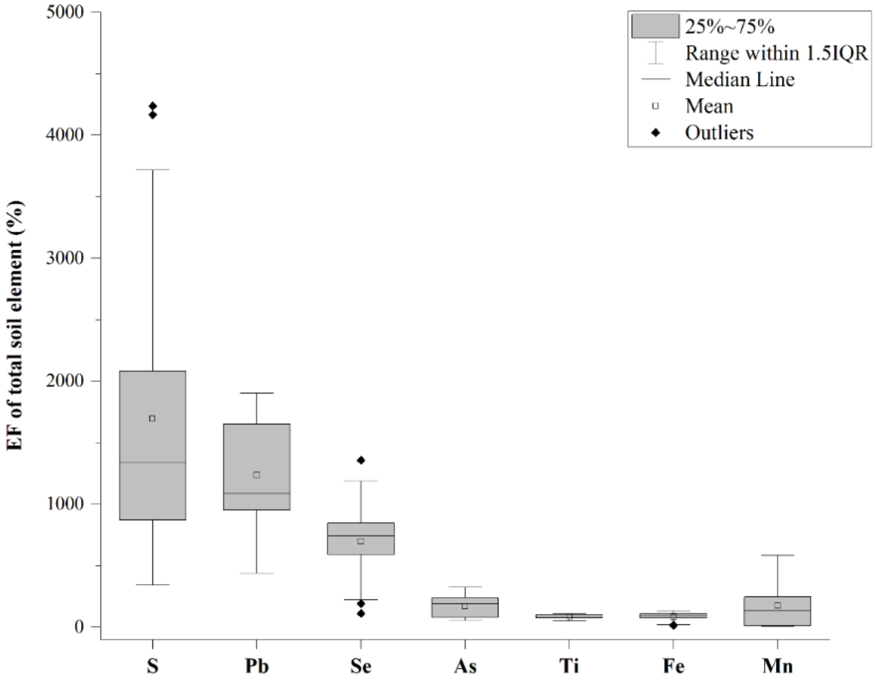

Supplementary Figure 17. Enrichment factors with respect to parent rock in Kohala soils for sulfur (S), lead (Pb), selenium (Se), arsenic (As), titanium (Ti), iron (Fe), and manganese (Mn). S and Pb are elements for which the atmosphere is a main source to Kohala soils[73,74], while Fe, Ti, and Mn are elements for which parent rock weathering dominates the inputs in Kohala soils[73.]

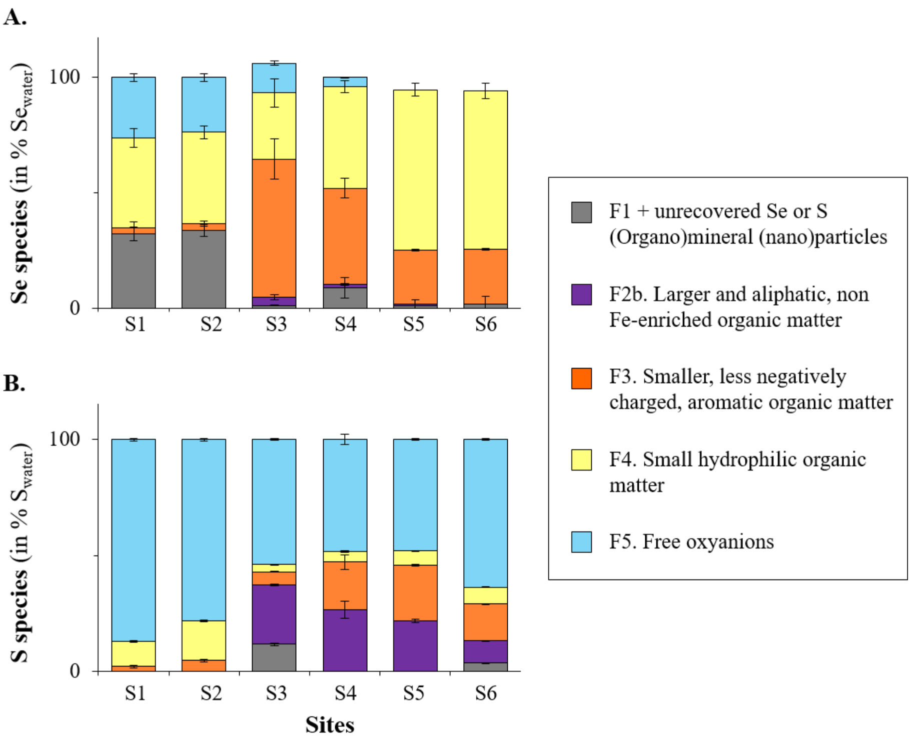

Supplementary Figure 18. Speciation of selenium (Se; panel A) and sulfur (S; panel B) in ultrapure water extracts of Kohala topsoils (0-10 cm). For all presented data, the error bar represents the standard deviation, which considers i) the standard deviation obtained during the SEC peak deconvolution; ii) the standard deviation of topsoil Se or topsoil S concentrations that were determined in triplicate.

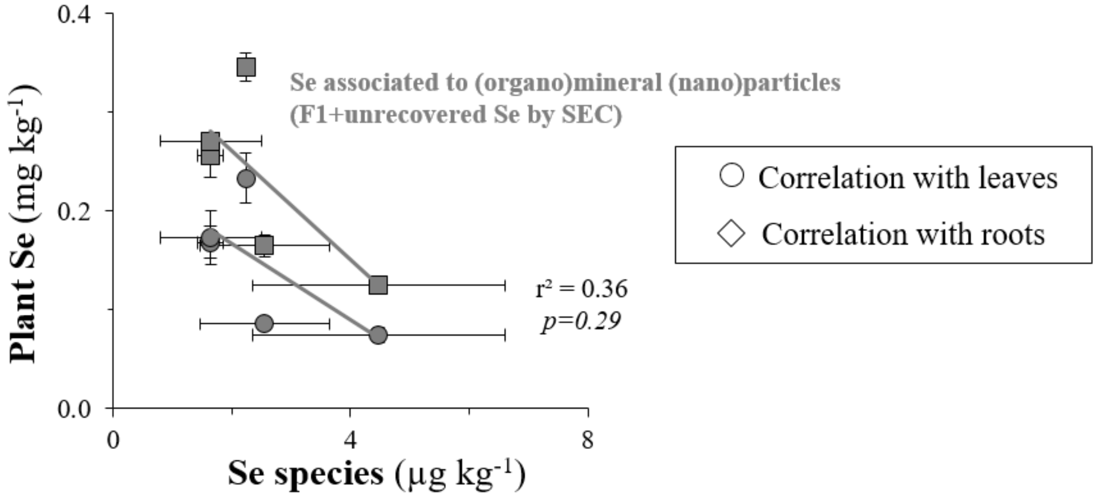

Supplementary Figure 19. 2-tailed Spearman correlation between the concentrations of selenium (Se) in plant materials (y-axis) and the concentrations of Se associated to (organo)mineral (nano)particles in water extracts of Kohala 0-10cm-topsoils (x-axis), when excluding site S3. Se associated to (organo)mineral (nano)particles includes Se quantified in SEC fraction F1 plus Se unrecovered by SEC because >20 nm.

Supplementary Method 1. Obtained data for certified reference materials

Supplementary Table 6. Measured and certified elemental concentrations (average and standard deviation) for the rock and soils certified reference materials (CRMs), validating the digestion procedure and subsequent ICP-MS analysis used to quantify elements in Kohala soils and parent rock.

	**[element]$_{measured}$** (mg kg^{-1})		**[element]$_{certified}$** (mg kg^{-1})		**Recovery**[a] (%)		**Error**[b] (%)
	av.[c]	sd.[d]	av.[c]	sd.[d]	av.[c]	sd.[d]	av.[c]
**NIST SRM-688 Basalt rock** (n=6 replicates)							
Iron (Fe)	71959	*1434*	72388	*21*	**99**	*2*	**-1**
Manganese (Mn)	920	29	1293	15	71	2	**-29**
Copper (Cu)	82	*9*	96		**85**	*10*	**-15**
Zinc (Zn)	60	*5*	58		**103**	*8*	**3**
Arsenic (As)	2.6	*0.1*	x		x		**x**
Selenium (Se)	<D.L.		x		x		**x**
Lead (Pb)	3.1	*0.3*	3.3	*0.2*	**95**	*11*	**-5**
**Sigma-Aldrich CRM-044 Silt Loam 1** (n=6 replicates)							
Iron (Fe)	3213	*169*	3180	*284*	**102**	*13*	**32**
Manganese (Mn)	231	8	204	14	113	9	**13**
Copper (Cu)	54	*4*	64	*2*	**84**	*8*	**-16**
Zinc (Zn)	124	*8*	136	*4*	**92**	*7*	**-8**
Arsenic (As)	48	*4*	52	*7*	**92**	*15*	**-8**
Selenium (Se)	75	*7*	81	*7*	**93**	*12*	**-7**
Lead (Pb)	79	*4*	78	*3*	**101**	*6*	**-12**
**NIST SRM-2709a San Joaquin soil** (n=4 replicates)							
Iron (Fe)	33045	1131	33600	700	**98**	4	**-2**
Manganese (Mn)	536	10	529	18	101	4	1
Copper (Cu)	30	1	34	1	**88**	2	**-12**
Zinc (Zn)	102	8	103	4	**99**	8	**-1**
Arsenic (As)	10.0	0.1	10.5	0.3	**95**	3	**-5**
Selenium (Se)	1.4	0.3	1.5		**96**	18	**-4**
Lead (Pb)	16.4	0.4	17.3	0.1	**95**	2	**-5**
**GBW 07405 Chinese Yellow-red soil** (n=4 replicates)							
Iron (Fe)	88685	901	93536	935	**95**	1	**-5**
Manganese (Mn)	1401	40	1360	27	**103**	4	**3**
Copper (Cu)	154	13	144	3	**107**	9	**7**
Zinc (Zn)	544	10	494	10	**110**	3	**10**
Arsenic (As)	404	3	412	8	**98**	2	**-2**
Selenium (Se)	1.7	0.3	1.6	0.1	**106**	10	**16**
Lead (Pb)	585	35	552	17	**106**	7	**6**

[a]Recoveries were calculated according to equation (2) given on next page; [b]Errors were calculated according to equation (3) given on next page; [c]av.: average value, which results from the digestion of the rock and soil CRMs in 4-6 replicates and the element quantification in each digestion replicate (with the ICP-MS/MS acquisition replicate being set to 3); [d]sd.: standard deviation value, which includes the standard deviation value associated to the element concentration obtained in the digestion replicate and the standard deviation value associated to the ICP-MS/MS acquisition being performed in triplicate for each digestion replicate. Note that for the recovery, the provided standard deviation value also includes the standard deviation value associated to the certified element concentrations (when this value is available).

$$\text{Recovery} = \frac{[\text{element}]_{\text{measured}}}{[\text{element}]_{\text{certified}}} \times 100 \qquad (2)$$

$$\text{Error} = \frac{([\text{element}]_{\text{measured}} - [\text{element}]_{\text{certified}})}{[\text{element}]_{\text{certified}}} \times 100 \qquad (3)$$

with: $[\text{element}]_{\text{measured}}$ representing the element concentration measured in the certified reference material and $[\text{element}]_{\text{certified}}$ representing the certified concentrations of the element in the certified reference materials

Supplementary Table 7. Measured and certified element concentrations (average and standard deviation) for the two plant certified reference materials (CRMs), validating the digestion procedure and subsequent ICP-MS/MS analysis used to quantify arsenic (As), sulfur (S), and selenium (Se) in Kohala plant leaves and roots.

	**[element]$_{\text{measured}}$** (mg kg^{-1})	**[element]$_{\text{certified}}$** (µg L^{-1})	**Recovery**[a] (%)	**Error**[b] (%)
	**av.**[c] ± sd[d]	**av.**[c] ± sd[d]	**av.**[c] ± sd[d]	**av.**[c]
**NIST SRM-1515 Apple leaves** (n=3 replicates)				
Arsenic (As)	**0.032** ± 0.002	**0.038**	**79.6** ± 0.6	**-20**
Sulfur (S)	**1575** ± 47	**1800**	**88** ± 3	**-12**
Selenium (Se)	**0.048** ± 0.003	**0.05**	**96** ± 7	**-4**
**NCS-DC73349 Bush branches** (n=3 replicates)				
Arsenic (As)	**0.997** ± 0.003	**1.25**	**79.7** ± 0.2	**-20**
Sulfur (S)	**6304** ± 96	**7300**	**86** ± 1	**-14**
Selenium (Se)	**0.127** ± 0.006	**0.13**	**97** ± 5	**-3**

[a]Recoveries were calculated according to equation (2); [b]Errors were calculated according to equation (3); [c]av.: average value, which results from the digestion of the plant CRMs in triplicate and the element quantification in each digestion triplicate (with the ICP-MS/MS acquisition replicate being set to 3); [d]sd.: standard deviation value, which includes the standard deviation value associated to the element concentration obtained in the digestion triplicate and the standard deviation value associated to the ICP-MS/MS acquisition being performed in triplicate for each digestion replicate. Note that for the recovery, the provided standard deviation value also includes the standard deviation value associated to the certified element concentrations (when this value is available).

Supplementary Table 8. Measured and certified elemental concentrations (average and standard deviation, n= 4) for two liquid CRMs (SRM 1643f and TMDA-51.2) diluted in the used extractant (water and NaOH), validating the procedure of quantification of elements in soil extracts by ICP-MS/MS.

	**NIST SRM-1643f freshwater** (n=4 replicates)				NRCAN TMDA-51.2 (n=4 replicates)			
	**[element]$_{measured}$** (µg L^{-1})	**[element]$_{certified}$** (µg L^{-1})	**Recovery**[a] (%)	**Error**[b] (%)	**[element]$_{measured}$** (µg L^{-1})	**[element]$_{certified}$** (µg L^{-1})	**Recovery**[a] (%)	**Error**[b] (%)
	**av.**[c] ± sd[d]	**av.**[c] ± sd[d]	**av.**[c] ± sd[d]	**av.**[c]	**av.**[c] ± sd[d]	**av.**[c] ± sd[d]	**av.**[c] ± sd[d]	**av.**[c]
**Diluted in NaOH**								
Fe	**85** ± 6	**93** ± 1	**92** ± 6	***-8***	**108** ± 22	**93** ± 1	**97** ± 30	**-3**
Cu	**20** ± 3	**21.7** ± 0.7	**90** ± 15	***-10***	**92** ± 12	**91** ± 10	**101** ± 17	**1**
Zn	**76** ± 1	**74** ± 2	**102** ± 2	***2***	**122** ± 1	**106** ± 15	**116** ± 16	**16**
As	**57** ± 3	**57.4** ± 0.4	**99** ± 6	***-1***	**16** ± 1	**15** ± 3	**106** ± 24	**6**
Se	**11** ± 1	**11.7** ± 0.1	**97** ± 9	***-3***	**12** ± 1	**12** ± 3	**102** ± 27	**2**
Pb	**19** ± 1	**18.5** ± 0.1	**102** ± 6	***2***	**72** ± 8	**73** ± 11	**98** ± 18	**-2**
**Diluted in ultrapure water**								
Fe	**111** ± 1	**111** ± 26	**100** ± 23	***0***	**94** ± 1	**93** ± 1	**101** ± 1	**1**
Cu	**18.4** ± 0.4	**21.7** ± 0.7	**85** ± 3	***-15***	**87** ± 1	**91** ± 10	**96** ± 11	**-4**
Zn	**66** ± 6	**74** ± 2	**89** ± 8	***-11***	**90** ± 8	**106** ± 15	**85** ± 14	**-15**
As	**54** ± 1	**57.4** ± 0.4	**95** ± 2	***-5***	**14.0** ± 0.5	**15** ± 3	**92** ± 21	**-8**
Se	**11.1** ± 0.4	**11.7** ± 0.1	**95** ± 4	***-5***	**11.2** ± 0.2	**12** ± 3	**93** ± 23	**-7**
Pb	**17.6** ± 0.4	**18.5** ± 0.1	**95** ± 2	***-5***	**69** ± 2	**73** ± 11	**95** ± 14	**-5**

[a]Recoveries were calculated according to equation (2); [b]Errors were calculated according to equation (3); [c]av.: average value, which results from the digestion of the plant CRMs in triplicate and the element quantification in each digestion triplicate (with the ICP-MS/MS acquisition replicate being set to 3); [d]sd.: standard deviation value, which includes the standard deviation value associated to the element concentration obtained in the digestion triplicate and the standard deviation value associated to the ICP-MS/MS acquisition being performed in triplicate for each digestion replicate. Note that for the recovery, the provided standard deviation value also includes the standard deviation value associated to the certified element concentrations (when this value is available).

Supplementary Method 2. Comparison of water extraction efficiencies obtained with different solid/liquid ratios

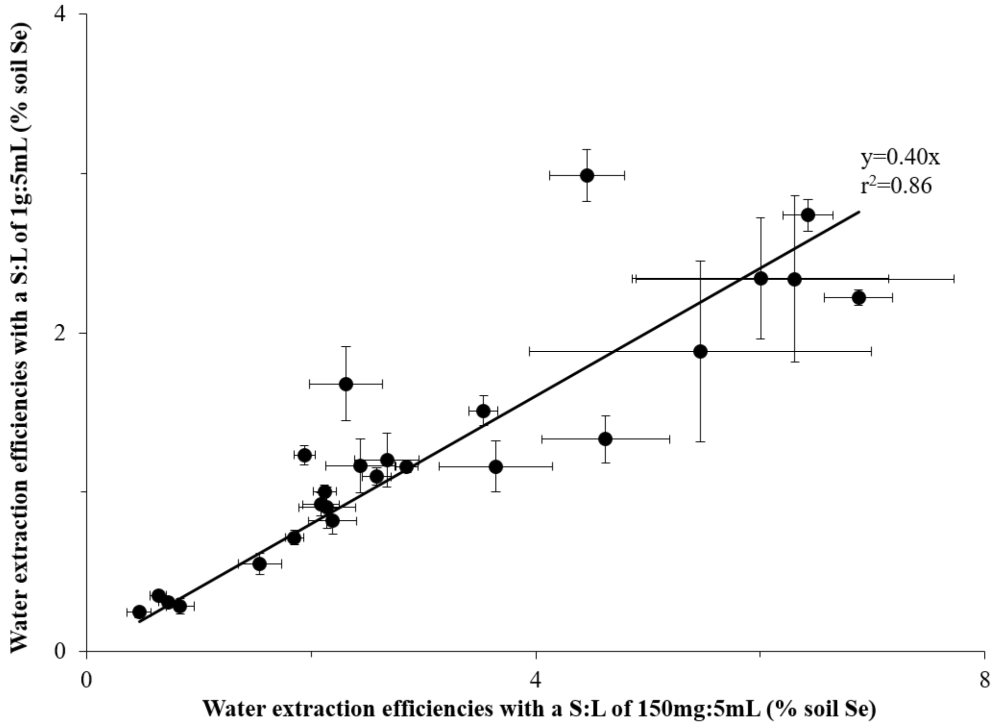

Supplementary Figure 20. 2-tailed Spearman correlation ($p<0.001$) between the efficiencies of ultrapure water extraction for selenium (Se, in % of soil Se concentration) obtained with a solid:liquid (S:L) ratio of 1g:5mL (y-axis) and a S:L ratio of 150mg:5mL (x-axis). For both y and x parameters, the error bar represents the standard deviation, which considers 1) the standard deviation associated to the determination of Se concentration in the extract in triplicate (ICP-MS/MS acquisition replicate being set to 3); and 2) the standard deviation associated to the determination of Se concentration in the soil determined in triplicate (digestion triplicate).

Supplementary Method 3. On-line isotope dilution calculation including interferences and mass bias corrections

From the post UV-detector addition of ^{78}Se(IV) and the acquisition of Se and bromine (Br) isotopes by ICP-MS/MS during the SEC-UV-ICP-MS/MS analysis of all Kohala soil extracts, the Se mass flow chromatogram (M_f), which enables to obtain the Se amount in each SEC peak, was determined following the steps and equation described below.

Step 1. Interferences corrections.

For each data point along the SEC elution, the intensities of ^{78}Se and ^{80}Se were corrected for $^{77}Se^{1}H^{+}$ and $^{79}Br^{1}H^{+}$ interferences that are formed in the plasma using equations (5) to (7)

$$^{77}I_c = {}^{77}I - (fSe \,.\, {}^{76}I) \quad (4)$$
$$^{78}I_c = {}^{78}I - (fSe.\, {}^{77}I_c) \quad (5)$$
$$^{79}I_c = {}^{79}I - (fSe \,.\, {}^{78}I_c) \quad (6)$$
$$^{80}I_c = {}^{80}I - (fBr \,.\, {}^{79}I_c) \quad (7)$$

^{x}I is the gross signal intensity measured at the *m/z* x (x=77, 78 79, or 80) with 77, 78, 80 being Se isotopes and 79 being a Br isotope. $^{x}I_c$ (x=77, 78 79, or 80) is the signal intensity of the Se or Br isotope after correction for SeH^+ or BrH^+ interferences. *fSe* is the factor of SeH^+ formation determined by measuring the *m/z* ratio 83/82 ($^{82}Se^{1}H^{+}/^{82}Se$) in a 10 µg L^{-1} Se standard of natural abundance by ICP-MS/MS (see Supplementary Table 9). *fBr* is the factor of BrH^+ formation determined by measuring the *m/z* ratio 80/79 ($^{81}Br^{1}H^{+}/^{81}Br$) ratios in a 10 µg L^{-1} Br standard of natural abundance). Supplementary Table 9 provides the values of *fSe* and *fBr* obtained each day before starting the SEC-UV-ICP-MS/MS run, which were used to calculate the Se mass flow chromatograms for the samples of the corresponding run. It worth to point that there is little variation of *fSe* and *fBr* between days of analysis.

Supplementary Table 9. Values of selenium hydridation (*fSe*, in %) and bromine hydridation (*fBr*, in %) factors as well as of mass bias (*K*, in %) obtained for the different performed SEC-UV-ICP-MS/MS runs and used for the on-line isotopic dilution calculation. *fSe, fBr and K* were determined by analyzing in triplicate using the ICP-MS/MS a 10 µg L^{-1} Se standard of natural abundance and a 10 µg L^{-1} Br standard of natural abundance the day before starting the SEC-UV-ICP-MS/MS runs.

	***fSe* (%)**	***fBr* (%)**	***K* (%)**
	**av.**[a] ± sd[b] (RSD[c])	**av.**[a] ± sd[b] (RSD[c])	**av.**[a] ± sd[b] (RSD[c])
**Run 1**	**0.19** ± 0.01 (5%)	**0.40** ± 0.02(5%)	**0.032** ± 0.001 (3%)
**Run 2**	**0.189** ± 0.005 (3%)	**0.44** ± 0.01 (2%)	**0.027** ± 0.001 (4%)
**Run 3**	**0.19** ± 0.01(5%)	**0.42** ± 0.02 (5%)	**0.030** ± 0.001 (3%)
**Run 4**	**0.17** ± 0.01(5%)	**0.41** ± 0.03(8%)	**0.027** ± 0.001 (4%)

[a]av.: average value, considering the preparation of the natural abundance Se and Br standards in triplicate and the analysis in triplicate (ICP-MS/MS acquisition replicate set to 3) of each standards prepared three times; [b]sd : standard deviation value, which includes the standard deviation value associated to the preparation and analysis of the natural abundance Se and Br standards in triplicate and the standard deviation value associated to the ICP-MS/MS acquisition being performed in triplicate for each prepared standard triplicate; [c]RSD : relative standard deviation.

Step 2. Mass bias corrections.

For each data point along the SEC elution, the ratio $^{80}Se/^{78}Se$ ($R_{sample,\ measured}$) was calculated using the corrected intensities of ^{78}Se and ^{80}Se, and this ratio was corrected for mass bias using the equation (8).

$$R_{sample,\ corrected} = R_{sample,\ measured} \cdot e^{-K \cdot \Delta M_{80/78}} \qquad (8)$$

$R_{sample,\ measured}$ is the $^{80}Se/^{78}Se$ ratio calculated after correcting ^{78}Se and ^{80}Se intensities for interferences as showin in equation (4) to (7). $\Delta M_{80/78}$ is the mass difference between ^{80}Se and ^{78}Se ($\Delta M_{80/78}$ = 2). *K* is the mass bias factor determined from the analyses of a 10 µg L^{-1} natural abundance Se standards. More precisely, to determine *K*, the Napierian logarithm of the relative error of the experimentally measured isotopic ratio $^{80}Se/^{78}Se$ ratio in the natural abundance Se standard ($R_{experimental}$) with respect to the theoretical natural abundance ratio ($R_{theoretical}$) is plot against the mass difference (ΔM) between the isotope of reference (here ^{78}Se) and the other monitored isotopes (as shown in Supplementary Figure 21)[10]. The result is a linear relationship and the mass bias factor (*K*) derived from the slope of the regression line. Supplementary Table 9 provides the values of *K* obtained each day before starting the SEC-UV-ICP-MS/MS run, which were used to calculate the Se mass flow chromatograms for the

samples of the corresponding run. It worth to point that there is little variation of K between days of analysis

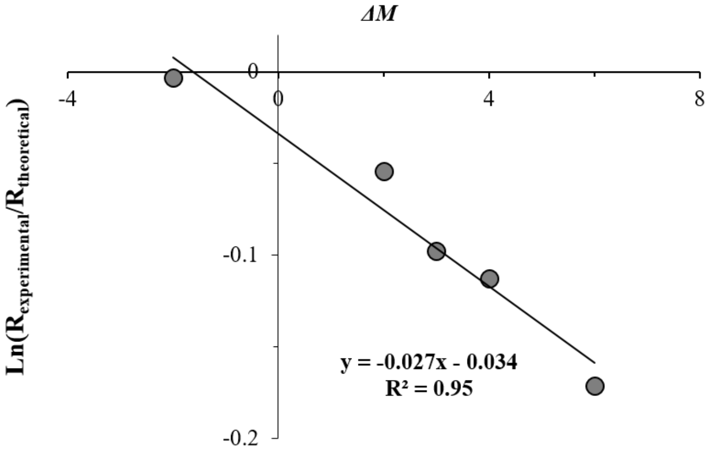

Supplementary Figure 21. Determination of *K* from the linear relationship between Ln ($R_{experimental}/R_{theoretical}$) and the mass difference (*ΔM*) between the monitored isotopes (i.e., ^{76}Se, ^{77}Se, ^{80}Se and ^{82}Se) and the ^{78}Se reference isotope, from the analyses of a 10 µg L^{-1} natural abundance Se standards. The K value obtained in this analysis is of 2.7 %.

Step 3. Isotope dilution calculation to create the mass flow chromatogram

The Se mass flow chromatogram is created by calculating M_s (unit, ng min^{-1}) for each data point along the SEC elution using equation (9) that was adapted from Sariego Muňiz et al.[55] equations.

$$M_s = C_{sp} \, . \, d_{sp} \, . \, f_{sp} \, . \frac{AW_s}{AW_{sp}} \, . \frac{A_{sp}^{78}}{A_s^{80}} \, . \frac{(R_m - R_{sp})}{(1 - R_m . R_{sp})} \qquad (9)$$

C_{sp} is the concentration of ^{78}Se(IV) in the spike solution added post-UV detector (in ng g^{-1}), which was adjust between samples; d_{sp} is the density of the ^{78}Se(IV) spike solution (g mL^{-1}), i.e., of 1 g mL^{-1}; f_{sp} is the flow rate to which the ^{78}Se(IV) spike solution is added post-UV detector (mL min^{-1}), i.e., of 0.068 mL min^{-1} in this study; AW_s is the atomic weight of selenium in the sample, i.e., 79.04; AW_{sp} is the atomic weight of selenium in the ^{78}Se(IV) spike solution, i.e., 77.73 in this study; A^{78}_{sp} is the abundance of ^{78}Se in the added^{78}Se(IV) spike solution, i.e., of 99.50 in this study; A^{80}_{s} is the abundance of ^{80}Se in the sample, i.e., natural abundance which is of 49.61%; R_m is the ^{80}Se/^{78}Se ratio after interferences and mass bias corrections (see equations (4) to (8)); R_{sp} is the ^{80}Se/^{78}Se ratio in the added^{78}Se(IV) spike solution, i.e., of 0.0033 in this study.

Supplementary References

1. Kang, Y., Yamada, H., Kyuma, K., Hattori, T. & Kigasawa, S. Selenium in soil humic acid. *Soil Science and Plant Nutrition* **37**, 241–248 (1991).
2. Séby, F., Potin-Gautier, M., Lespés, G. & Astruc, M. Selenium speciation in soils after alkaline extraction. *The Science of the Total Environment* **207**, 81–90 (1997).
3. Martens, D. A. & Suarez, D. L. Selenium speciation of soil/sediment determined with sequential extractions and Hydride Generation Atomic Absorption Spectrophotometry. *Environmental Science and Technology* **31**, 133–139 (1997).
4. Zhang, Y. & Frankenberger jr., W. T. Determination of selenium fractionation and speciation in wetland sediments by parallel extraction. *International Journal of Environmental Analytical Chemistry* **83**, 315–326 (2003).
5. O. Darcheville *et al.* Aqueous, solid and gaseous partitioning of selenium in an oxic sandy soil under different microbiological states. *Journal of Environmental Radioactivity* **99**, 981–992 (2008).
6. Tolu, J., Le Hécho, I., Bueno, M., Thiry, Y. & Potin-Gautier, M. Selenium speciation analysis at trace level in soils. *Anal. Chim. Acta* **684**, 126–133 (2011).
7. Qin, H., Zhu, J. & Su, H. Selenium fractions in organic matter from Se-rich soils and weathered stone coal in selenosis areas of China. *Chemosphere* **86**, 626–633 (2012).
8. Wang, J. *et al.* Speciation, Distribution, and Bioavailability of Soil Selenium in the Tibetan Plateau Kashin–Beck Disease Area—A Case Study in Songpan County, Sichuan Province, China. *Biol. Trace Elem. Res.* **156**, 367–375 (2013).
9. Tolu, J. *et al.* Distribution and speciation of ambient selenium in contrasted soils, from mineral to organic rich. *Sci. Total Environ.* **479–480**, 93–101 (2014).
10. Tolu, J. *et al.* A new methodology involving stable isotope tracer to compare simultaneously short- and long-term selenium mobility in soils. *Anal. Bioanal. Chem.* **406**, 1221–1231 (2014).
11. Martens, D. A. & Suarez, D. L. Selenium speciation of marine shales, alluvial soils, and evaporation basin soils of California. *Journal of Environmental Quality* **26**, 424–432 (1997).
12. Wright, M. T., Parker, D. R. & Amrhein, C. Critical Evaluation of the Ability of Sequential Extraction Procedures To Quantify Discrete Forms of Selenium in Sediments and Soils. *Environ. Sci. Technol.* **37**, 4709–4716 (2003).
13. D. J. Ashworth, J. Moore, & G. Shaw. Effects of soil type, moisture content, redox potential and methyl bromide fumigation on K_d values of radio-selenium in soil. *Journal of Environmental Radioactivity* **99**, 1136–1142 (2008).
14. Y. Nakamaru, K. Tagami, & S. Uchiga. Distribution coefficient of selenium in Japanese agricultural soils. *Chemosphere* **58**, 1347–1354 (2005).
15. Qin, H.-B. *et al.* Selenium speciation in seleniferous agricultural soils under different cropping systems using sequential extraction and X-ray absorption spectroscopy. *Environ. Pollut.* **225**, 361–369 (2017).
16. Kulp, T. R. & Pratt, L. M. Speciation and weathering of selenium in Upper Cretaceous chalk and shale from South Dakota and Wyoming, USA. *Geochimica et Cosmochimica Acta* **68**, 3687–3701 (2004).
17. Sharmasarkar, S. & Vance, G. F. Extraction and distribution of soil organic and inorganic selenium in coal mine environments of Wyoming, USA. *Environ. Geol.* **29**, 17–22 (1997).
18. Dhillon, K. S., Dhillon, S. K. & Pareek, N. Distribution and bioavailability of selenium fractions in some seleniferous soils of Punjab, India: (Verteilung und Bioverfügbarkeit von Selenfraktionen in

einigen Selen haltigen Böden der Region Punjab, Indien). *Archives of Agronomy and Soil Science* **51**, 633–643 (2005).
19. Favorito, J. E., Luxton, T. P., Eick, M. J. & Grossl, P. R. Selenium speciation in phosphate mine soils and evaluation of a sequential extraction procedure using XAFS. *Environ. Pollut.* **229**, 911–921 (2017).
20. Coppin, F., Chabroullet, C., Martin-Garin, A., Balesdent, J. & Gaudet, J. P. Methodological approach to assess the effect of soil ageing on selenium behavior: first results concerning mobility and solid fractionation of selenium. *Biol. Fertil. Soils* **42**, 379–386 (2006).
21. Supriatin, S., Weng, L. & Comans, R. N. J. Selenium speciation and extractability in Dutch agricultural soils. *Sci. Total Environ.* **532**, 368–382 (2015).
22. Supriatin, S., Weng, L. & Comans, R. N. J. Selenium-rich dissolved organic matter determines selenium uptake in wheat grown on Low-selenium arable land soils. *Plant Soil* **408**, 73–94 (2016).
23. Weng, L., Vega, F. A., Supriatin, S., Bussink, W. & Van Riemsdijk, W. H. Speciation of Se and DOC in soil solution and their relation to Se bioavailability. *Environmental Science and Technology* **45**, 262–267 (2011).
24. Wang, S. *et al.* Selenium fractionation and speciation in agriculture soils and accumulation in corn (Zea mays L.) under field conditions in Shaanxi Province, China. *Sci. Total Environ.* **427–428**, 159–164 (2012).
25. Chao, T. T. & Sanzolone, R. F. Fractionation of soil selenium by sequential partial dissolution. *Soil Science Society of America Journal* **53**, 385–392 (1989).
26. Zhang, Y. & Moore, J. N. Selenium fractionation and speciation in a wetland system. *Environmental Science and Technology* **30**, 2613–2619 (1996).
27. K. S. Dhillon & S. K. Dhillon. Adsorption-desorption reactions of selenium in some soils of India. *Geoderma* **93**, 19–31 (1999).
28. Wang, M. C. & Chen, H. M. Forms and distribution of selenium at different depths and among particle size fractions of three Taiwan soils. *Chemosphere* **52**, 585–593 (2003).
29. Gao, S., Tanii, K. K., Peters, D. W. & Herbel, M. J. Water selenium speciation and sediment fractionation in a California flow-through wetland system. *Journal of Environmental Quality* **29**, 1275–1283 (2000).
30. Mathers, A. W. *et al.* Determining the fate of selenium in wheat biofortification: an isotopically labelled field trial study. *Plant Soil* **420**, 61–77 (2017).
31. da Silva Junior, E. C. *et al.* Geochemistry of selenium, barium, and iodine in representative soils of the Brazilian Amazon rainforest. *Science of The Total Environment* **828**, 154426 (2022).
32. Gil-García, C., Tagami, K., Uchida, S., Rigol, A. & Vidal, M. New best estimates for radionuclide solid-liquid distribution coefficients in soils. Part 3: Miscellany of radionuclides (Cd, Co, Ni, Zn, I, Se, Sb, Pu, Am, and others). *Journal of Environmental Radioactivity* **100**, 704–715 (2009).
33. Sheppard, S. C. Robust Prediction of Kd from Soil Properties for Environmental Assessment. *Human and Ecological Risk Assessment* **17**, 263–279 (2011).
34. Ponce de León, C. A., DeNicola, K., Montes-Bayón, M. & Caruso, J. A. Sequential extractions of selenium soils from Stewart Lake: total selenium and speciation measurements with ICP-MS detection. *Journal of Environmental Monitoring* **5**, 435–440 (2003).
35. Tokunaga, T. K. *et al.* Soil selenium fractionation, depth profiles and time trends in a vegetated site at Kesterson Reservoir. *Water, Air, and Soil Pollution* **57/58**, 31–41 (1991).
36. Stroud, J. L. *et al.* Soil factors affecting selenium concentration in wheat grain and the fate and speciation of Se fertilisers applied to soil. *Plant Soil* **332**, 19–30 (2010).

37. Bujdoš, M., Kubová, J. & Streško, V. Problems of selenium fractionation in soils rich in organic matter. *Analytica Chimica Acta* **408**, 103–109 (2000).
38. Bujdoš, M., Mul'ová, A., Kubová, J. & Medved', J. Selenium fractionation and speciation in rocks, soils, waters and plants in polluted surface mine environment. *Environmental Geology* **47**, 353–360 (2005).
39. Kang, Y., Yamada, H., Kyuma, K. & Hattori, T. Speciation of selenium in soil. *Soil Science and Plant Nutrition* **39**, 331–337 (1993).
40. Gustafsson, J. P. & Johnsson, L. The Association between Selenium and Humic Substances in Forested Ecosystems-Laboratory Evidence. *Appl. Organomet. Chem.* **8**, 141–147 (1994).
41. Olk, D. C. *et al.* Environmental and Agricultural Relevance of Humic Fractions Extracted by Alkali from Soils and Natural Waters. *J. Environ. Qual.* **48**, 217 (2019).
42. Abrams, M. M., Burau, R. G. & Zasoski, R. J. Organic selenium distribution in selected California soils. *Soil Science Society of America Journal* **54**, 979–982 (1990).
43. GUSTAFSSON, J. P. & JOHNSSON, L. Selenium retention in the organic matter of Swedish forest soils. *Journal of Soil Science* **43**, 461–472 (1992).
44. Jackson, B. P. & Miller, W. P. Effectiveness of Phosphate and Hydroxide for Desorption of Arsenic and Selenium Species from Iron Oxides. *Soil Sci. Soc. Am. J.* **64**, 1616–1622 (2000).
45. Helfenstein, J. *et al.* Soil Phosphorus Exchange as Affected by Drying-Rewetting of Three Soils From a Hawaiian Climatic Gradient. *Front. Soil Sci.* **1**, 738464 (2021).
46. Martin, D. P., Seiter, J. M., Lafferty, B. J. & Bednar, A. J. Exploring the ability of cations to facilitate binding between inorganic oxyanions and humic acid. *Chemosphere* **166**, 192–196 (2017).
47. Schmitt, D., Müller, M. B. & Frimmel, F. H. Metal Distribution in Different Size Fractions of Natural Organic Matter. *Acta hydrochimica et hydrobiologica* **28**, 400–410 (2001).
48. Jackson, B. P., Ranville, J. F., Bertsch, P. M. & Sowder, A. G. Characterization of Colloidal and Humic-Bound Ni and U in the "Dissolved" Fraction of Contaminated Sediment Extracts. *Environ. Sci. Technol.* **39**, 2478–2485 (2005).
49. Huber, S. A., Balz, A., Abert, M. & Pronk, W. Characterisation of aquatic humic and non-humic matter with size-exclusion chromatography – organic carbon detection – organic nitrogen detection (LC-OCD-OND). *Water Res.* **45**, 879–885 (2011).
50. Liu, G. & Cai, Y. Complexation of arsenite with dissolved organic matter: Conditional distribution coefficients and apparent stability constants. *Chemosphere* **81**, 890–896 (2010).
51. Wu, F. C., Evans, R. D., Dillon, P. J. & Cai, Y. R. Rapid quantification of humic and fulvic acids by HPLC in natural waters. *Appl. Geochem.* **22**, 1598–1605 (2007).
52. Kozyatnyk, I., Bouchet, S., Björn, E. & Haglund, P. Fractionation and size-distribution of metal and metalloid contaminants in a polluted groundwater rich in dissolved organic matter. *J. Hazard. Mater.* **318**, 194–202 (2016).
53. Hawkes, J. A., Sjöberg, P. J. R., Bergquist, J. & Tranvik, L. J. Complexity of dissolved organic matter in the molecular size dimension: insights from coupled size exclusion chromatography electrospray ionisation mass spectrometry. *Faraday Discuss.* 10.1039.C8FD00222C (2019) doi:10.1039/C8FD00222C.
54. Dael, P. V., Davidsson, L., Muñoz-Box, R., Fay, L. B. & Barclay, D. Selenium absorption and retention from a selenite- or selenate-fortified milk-based formula in men measured by a stable-isotope technique. *Br. J. Nutr.* **85**, 157–163 (2001).

55. Sariego Muñiz, C., Marchante Gayón, J. M., García Alonso, J. I. & Sanz-Medel, A. Speciation of essential elements in human serum using anion-exchange chromatography coupled to post-column isotope dilution analysis with double focusing ICP-MS. *J. Anal. At. Spectrom.* **16**, 587–592 (2001).
56. Hinojosa Reyes, L., Marchante-Gayón, J. M., García Alonso, J. I. & Sanz-Medel, A. Quantitative speciation of selenium in human serum by affinity chromatography coupled to post-column isotope dilution analysis ICP-MS. *J. Anal. At. Spectrom.* **18**, 1210–1216 (2003).
57. Laborda, F., Jiménez-Lamana, J., Bolea, E. & Castillo, J. R. Selective identification, characterization and determination of dissolved silver(i) and silver nanoparticles based on single particle detection by inductively coupled plasma mass spectrometry. *J. Anal. At. Spectrom.* **26**, 1362 (2011).
58. Klučáková, M. Size and Charge Evaluation of Standard Humic and Fulvic Acids as Crucial Factors to Determine Their Environmental Behavior and Impact. *Front. Chem.* **6**, 235 (2018).
59. Loosli, F., Yi, Z., Wang, J. & Baalousha, M. Improved extraction efficiency of natural nanomaterials in soils to facilitate their characterization using a multimethod approach. *Sci. Total Environ.* **677**, 34–46 (2019).
60. Nguyen, D. N., Grybos, M., Rabiet, M. & Deluchat, V. Effect of extraction methods on mobilizable colloids and associated phosphorus from reservoir sediment. *Chemosphere* **284**, 131321 (2021).
61. Guénet, H. *et al.* Highlighting the wide variability in arsenic speciation in wetlands: A new insight into the control of the behavior of arsenic. *Geochim. Cosmochim. Acta* **203**, 284–302 (2017).
62. Neubauer, E. *et al.* The influence of pH on iron speciation in podzol extracts: Iron complexes with natural organic matter, and iron mineral nanoparticles. *Sci. Total Environ.* **461–462**, 108–116 (2013).
63. Regelink, I. C., Voegelin, A., Weng, L., Koopmans, G. F. & Comans, R. N. J. Characterization of Colloidal Fe from Soils Using Field-Flow Fractionation and Fe K-Edge X-ray Absorption Spectroscopy. *Environ. Sci. Technol.* **48**, 4307–4316 (2014).
64. Persson, L., Alsberg, T., Kiss, G. & Odham, G. On-line size-exclusion chromatography/electrospray ionisation mass spectrometry of aquatic humic and fulvic acids. *Rapid Commun. Mass Spectrom.* **14**, 286–292 (2000).
65. Siripinyanond, A., Barnes, R. M. & Amarasiriwardena, D. Flow field-flow fractionation-inductively coupled plasma mass spectrometry for sediment bound trace metal characterization. *J. Anal. At. Spectrom.* **17**, 1055–1064 (2002).
66. Poulin, B. A., Ryan, J. N. & Aiken, G. R. Effects of Iron on Optical Properties of Dissolved Organic Matter. *Environ. Sci. Technol.* **48**, 10098–10106 (2014).
67. Chin, Y.-Ping., Aiken, George. & O'Loughlin, Edward. Molecular Weight, Polydispersity, and Spectroscopic Properties of Aquatic Humic Substances. *Environ. Sci. Technol.* **28**, 1853–1858 (1994).
68. Fujii, M., Imaoka, A., Yoshimura, C. & Waite, T. D. Effects of Molecular Composition of Natural Organic Matter on Ferric Iron Complexation at Circumneutral pH. *Environ. Sci. Technol.* **48**, 4414–4424 (2014).
69. Dinu, M. I. Comparison of complexing ability of fulvic and humic acids in the aquatic environment with iron and zinc ions. *Water Resour.* **37**, 65–69 (2010).
70. Westergaard Strobel, B., Bernhoft, I. & Borggaard, O. K. Low-molecular-weight aliphatic carboxylic acids in soil solutions under different vegetations determined by capillary zone electrophoresis. *Plant Soil* **212**, 115–121 (1999).
71. Bolan, N. S. *et al.* Dissolved Organic Matter. *Adv. Agron.* **110**, 1–75 (2011).

72. Von Wandruszka, R., Schimpf, M., Hill, M. & Engebretson, R. Characterization of humic acid size fractions by SEC and MALS. *Org. Geochem.* **30**, 229–235 (1999).
73. Teutsch, N., Erel, Y., Halicz, L. & Chadwick, O. A. The influence of rainfall on metal concentration and behavior in the soil. *Geochim. Cosmochim. Acta* **63**, 3499–3511 (1999).
74. Bern, C. R., Chadwick, O. A., Kendall, C. & Pribil, M. J. Steep spatial gradients of volcanic and marine sulfur in Hawaiian rainfall and ecosystems. *Sci. Total Environ.* **514**, 250–260 (2015).
